# Nitrogen fixation and fertilization have similar effects on biomass allocation in nitrogen‐fixing plants

**DOI:** 10.1002/ece3.70309

**Published:** 2024-09-17

**Authors:** Duncan N. L. Menge, Amelia A. Wolf, Jennifer L. Funk, Steven S. Perakis, K. A. Carreras Pereira

**Affiliations:** ^1^ Department of Ecology, Evolution, and Environmental Biology Columbia University New York City New York USA; ^2^ Department of Integrative Biology University of Texas at Austin Austin Texas USA; ^3^ Department of Plant Sciences University of California, Davis Davis California USA; ^4^ U.S. Geological Survey, Forest and Rangeland Ecosystem Science Center Corvallis Oregon USA

**Keywords:** actinorhizal, legume, rhizobial, symbiosis

## Abstract

Plants adjust their allocation to different organs based on nutrient supply. In some plant species, symbioses with nitrogen‐fixing bacteria that live in root nodules provide an alternate pathway for nitrogen acquisition. Does access to nitrogen‐fixing bacteria modify plants' biomass allocation? We hypothesized that access to nitrogen‐fixing bacteria would have the same effect on allocation to aboveground versus belowground tissues as access to plentiful soil nitrogen. To test this hypothesis and related hypotheses about allocation to stems versus leaves and roots versus nodules, we conducted experiments with 15 species of nitrogen‐fixing plants in two separate greenhouses. In each, we grew seedlings with and without access to symbiotic bacteria across a wide gradient of soil nitrogen supply. As is common, uninoculated plants allocated relatively less biomass belowground when they had more soil nitrogen. As we hypothesized, nitrogen fixation had a similar effect as the highest level of fertilization on allocation aboveground versus belowground. Both nitrogen fixation and high fertilization led to ~10% less biomass allocated belowground (~10% more aboveground) than the uninoculated, lowest fertilization treatment. Fertilization reduced allocation to nodules relative to roots. The responses for allocation of aboveground tissues to leaves versus stems were not as consistent across greenhouses or species as the other allocation trends, though more nitrogen fixation consistently led to relatively more allocation to leaves when soil nitrogen supply was low. Synthesis: Our results suggest that symbiotic nitrogen fixation causes seedlings to allocate relatively less biomass belowground, with potential implications for competition and carbon storage in early forest development.

## Graphical abstract

It has long been known that plants adjust allocation to different tissues based on supply of soil nitrogen, but the degree to which nitrogen supplied by symbiotic nitrogen‐fixing bacteria has similar effects on allocation is less well known. Using an experiment with 15 plant species with and without access to symbiotic nitrogen‐fixing bacteria, grown across a range of soil nitrogen supply, we isolated the effects of symbiotic nitrogen fixation versus soil nitrogen supply. We found that nitrogen fixation has similar effects on biomass allocation as nitrogen fertilization.

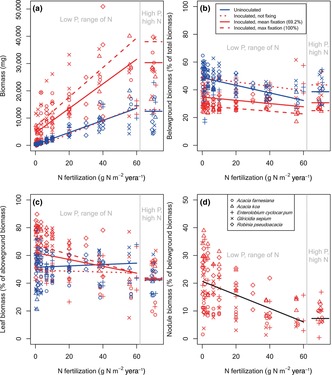

## 1 INTRODUCTION

Plants allocate biomass to different organs with different functions (Bazzaz & Grace, 1997). For example, leaves photosynthesize, stems provide structure and aid in light competition, and roots anchor plants to the ground and forage for nutrients and water (Poorter et al., 2012). Allocation to different tissues has important consequences ranging from life history to the global carbon cycle (Bazzaz & Grace, 1997; Iwasa, 2000). For example, stems persist longer and decompose slower than leaves or fine roots, so more biomass allocation to stems sustains carbon storage, with clear implications for global climate (Friend et al., 2014). In certain plants, root nodules house symbiotic bacteria that fix dinitrogen gas (Huss‐Danell, 1997; Sprent, 2009). As an additional source of nitrogen (N), N fixation could influence biomass allocation, but this has been much less explored than the effect of soil N.

It has long been known that plants adjust their allocation based on resource supply (Brenchley, 1916; Maximov & Yapp, 1929; Shirley, 1929). Decades of empirical work show that plants allocate more biomass belowground when in need of belowground resources, particularly nutrients (Brenchley, 1916; Chapin III, 1980; Ingestad & Agren, 1991; McCarthy & Enquist, 2007; Poorter et al., 2012; Poorter & Nagel, 2000). However, the degree of plasticity of biomass allocation varies widely across plants (Chapin III, 1980), and plants also have other ways to respond to nutrient limitation, such as altering stoichiometry within plant organs (Poorter et al., 2012). The physiological and genetic mechanisms underpinning how nutrient limitation alters belowground versus aboveground allocation are relatively well understood (Hermans et al., 2006; Poorter et al., 2012). Substantial theory using multiple approaches also supports the idea that nutrient limitation leads to greater allocation belowground (e.g., Bloom et al., 1985; Dybzinski et al., 2011; Ingestad & Agren, 1991; Poorter & Nagel, 2000; Reynolds & Pacala, 1993; Thornley, 1972; Wilson, 1988). These theoretical approaches range from optimality approaches that maximize growth rates (e.g., Bloom et al., 1985; Thornley, 1972) to evolutionarily stable strategy approaches that maximize fitness in a competitive context (e.g., Dybzinski et al., 2011).

Given the different functional roles of leaves versus stems and the different degrees of scaling with body size, a number of researchers have suggested dividing tissues into roots, leaves, and stems rather than simply roots and shoots (McCarthy & Enquist, 2007; Poorter & Nagel, 2000). Theoretical predictions for how nutrient addition affects allocation to leaves versus stems are less consistent than they are for aboveground versus belowground allocation. For example, Dybzinski et al. (2011) found that data from canopy‐level trees matched theoretical expectations from an evolutionarily stable strategy approach, which predicted that N addition leads to greater investment in wood as opposed to foliage. The proposed mechanism for their finding is that allocation to stems, which increases height, is more beneficial for light competition than packing additional leaves into an already full canopy (Dybzinski et al., 2011). However, a review across a broad array of plant types found different patterns at different degrees of N limitation. When N was scarce, increasing N availability led to greater investment in foliage as opposed to stems, but at moderate to high N availability, increasing N availability led to similar increases in both foliage and stems (Poorter et al., 2012). These studies focused on N rather than all nutrients, as do we, given its importance as a commonly limiting nutrient (LeBauer & Treseder, 2008) and given that our focus in this work is on the unique trait of N fixation.

In addition to acquiring N from the soil via their roots or mycorrhizal partners, certain species of plants can procure atmospheric N_2_ gas via symbioses with N‐fixing bacteria. These plants include most legumes (Fabaceae), which form “rhizobial” symbioses with rhizobia‐type bacteria (Sprent, 2009), and plants from eight other families that form “actinorhizal” symbioses with *Frankia*‐type bacteria (Huss‐Danell, 1997). Rhizobial plants are morphologically diverse, ranging from tropical trees to Mediterranean shrubs to arctic herbs (Sprent, 2009). They account for all N‐fixing crops and forage, such as soybean and alfalfa, and thus are indispensable for feeding humanity (Peoples et al., 2021). On the contrary, actinorhizal plants are almost entirely woody (Huss‐Danell, 1997). Actinorhizal plants comprise the majority of mid‐to‐high‐latitude N‐fixing tree symbioses, whereas rhizobial plants dominate the N‐fixing tree community at lower latitudes (Menge et al., 2017). Given their phylogenetic and morphological differences, it is conceivable that rhizobial versus actinorhizal groups allocate biomass differently. Alternatively, given their common ecological role as N fixers, perhaps their biomass allocation is similar. In both symbiotic types, dinitrogen gas is fixed in specialized root organs known as nodules whose sole purpose is to house symbiotic bacteria. N fixation in nodules can provide large quantities of N, raising interesting questions about biomass allocation. Does N fixation have similar effects on allocation as additional soil N such that fixing N leads to less allocation belowground? Or does the biomass required to build nodules simply replace the biomass that would have been used for roots, leading to similar aboveground versus belowground allocation? In addition to the structural cost of building nodules, N fixation also has metabolic costs (Gutschick, 1981; Tjepkema & Winship, 1980), but we focus on the structural costs, given our focus on biomass allocation.

A number of studies have examined the relative effects of soil N versus N fixation on biomass allocation in seedlings. Multiple studies with the actinorhizal genus *Alnus*—*A. incana* (Ingestad, 1980; Sellstedt, 1986; Sellstedt & Huss‐Danell, 1986), *A. viridis* (Markham & Zekveld, 2007), and *A. rubra* (Arnone III & Gordon, 1990)—found that inoculation had similar effects on aboveground versus belowground allocation as adding sufficient amounts of inorganic soil N to overcome N limitation of plant growth. These studies found that both inoculation and sufficient soil N led to relatively less biomass allocated belowground and relatively more aboveground biomass allocated to stems rather than leaves. Some of these studies also found that adding inorganic soil N decreased allocation to nodules (Ingestad, 1980; Markham & Zekveld, 2007), though another did not (Arnone III & Gordon, 1990). Dovrat et al. (2020) grew three species of herbaceous Mediterranean legumes and observed a different trend that suggests a role of inoculation itself: inoculation of plants that were already N‐sufficient led to relatively less biomass belowground. In an experiment with the tropical rhizobial N‐fixing tree *Pentaclethra macroloba*, Taylor and Menge (2021) found yet another trend: inoculated plants had similar aboveground versus belowground allocation as uninoculated plants, regardless of fertilization level, suggesting that nodule biomass simply replaced root biomass. Data from additional species are needed to determine if these distinct effects of inoculation on biomass allocation are broadly representative of the different taxonomic groups (actinorhizal trees vs. Mediterranean rhizobial shrubs vs. tropical rhizobial trees), the environmental conditions under which they were studied, or some other factor.

Here, we studied allocation of biomass to different tissues in 15 symbiotic plant taxa. We conducted two separate experiments, in two greenhouses, using similar manipulations in both experiments. We grew the plants across a wide range of soil N supply and, at the highest level of soil N supply, across two levels of soil P supply (see Section 2). We also manipulated the ability to fix N by inoculating half the plants with symbiotic bacteria. Within inoculated plants, the amount of fixation varied enough to allow us to statistically separate the effects of inoculation versus N fixation itself. We asked one basic question about three different allocation patterns: How do soil N supply, inoculation, and N fixation interact to affect allocation to (1) aboveground versus belowground tissues? (2) leaves versus stems, and (3) nodules versus roots? In the second experiment, we added a question: How do these allocation patterns differ between three different types of N‐fixing symbiosis: rhizobial tree species, actinorhizal tree species, and an agricultural herb (soybean)? We chose soybean as the agricultural herb because it is the largest provider of grain worldwide and it is a species in which N fixation has been well studied (Peoples et al., 2021).

Our overall hypotheses were that N fixation would have similar effects as soil N supply on allocation and that the effect of inoculation would be negligible aside from its effects on N fixation. Specifically, we tested the following hypotheses. (H1a) Both N fertilization and N fixation would decrease allocation to belowground tissues, as observed elsewhere for N fertilization in many nonfixing species (Brenchley, 1916; Ingestad & Agren, 1991; Poorter & Nagel, 2000) and for both N fertilization and inoculation (presumably through N fixation) with *Alnus* (Arnone III & Gordon, 1990; Ingestad, 1980; Markham & Zekveld, 2007; Sellstedt, 1986; Sellstedt & Huss‐Danell, 1986) and with Mediterranean shrubs (Dovrat et al., 2020). (H1b) Inoculation would act primarily through its effect on N fixation, that is, through increased N supply. In other words, an inoculated plant fixing a negligible amount of N would allocate biomass similarly to an uninoculated plant. For leaves versus stems, the theoretical work of Dybzinski et al. (2011) suggests greater allocation to stems relative to leaves with increasing soil N supply, but their theory was developed in the context of a closed canopy forest, whereas our experiments were in greenhouse conditions where additional leaves would also help capture more light. Therefore, we had competing hypotheses for leaves versus stems: both N fertilization and N fixation (H2a) increase, (H2b) have no effect on, or (H2c) decrease allocation to stems relative to leaves. For nodules, much past work has shown that N fertilization reduces allocation to nodules (Dovrat et al., 2018, 2020; Ingestad, 1980; Markham & Zekveld, 2007; McCulloch & Porder, 2021; Menge et al., 2015; Taylor & Menge, 2018; Uni et al., 2024), consistent with a facultative or incomplete downregulation strategy of N fixation (Hedin et al., 2009; Menge et al., 2009, 2015). However, some species in some conditions fix similar amounts of N with additional N fertilizer (Arnone III & Gordon, 1990; Binkley et al., 1994; Menge et al., 2023), consistent with an obligate N fixation strategy (Hedin et al., 2009; Menge et al., 2009, 2015). Following the bulk of evidence, we hypothesized (H3) a decrease in allocation to nodules with N fertilization.

## 2 METHODS

### 2.1 Greenhouses, growing conditions, and species

For our first experiment, in 2016–2017, we grew plants at Barnard College (New York, NY). For our second experiment, in 2018, we grew plants at UC Davis (Davis, CA). At Barnard, we used sharp sand (Gran‐i‐Grit) as a growing medium, whereas at UC Davis, we used a mixture of sharp sand and turface (calcine clay). Unless otherwise stated, the details described below applied to both experiments.

As is common, we studied seedlings rather than later stages of life history, for two main reasons. First, seedlings are an important life history stage, as the high mortality of seedlings means that biomass allocation in the seedling stage helps determine persistence into later stages. Second, seedlings are the only logistically feasible stage for studying the effects of inoculation. Furthermore, an investigation of the effects of N and P fertilization and N fixation (but not inoculation) on biomass allocation in an older life stage (4–5‐year‐old trees) of six of these species has been published recently (Carreras Pereira et al., 2023).

Prior to germination, we surface‐sterilized seeds and then grew plants in 10 × 10 cm pots. For inoculation, which was species‐specific, we used a slurry from crushed field‐collected nodules (for all plants grown at Barnard and some at UC Davis), cultured inoculum from the crushed nodules (for some plants grown at UC Davis), both the slurry and the culture (for some at UC Davis), or, in the case of soybean, a commercial strain (Table S1). For the slurry, ~15–30 mL of fresh nodules were surface‐sterilized and then crushed in a glass beaker with a glass rod. DI water was added to create a slurry of ~100–150 mL total volume. Half of the slurry was sterilized in an autoclave; half was not. Each plant of a given species received the same amount of slurry. The slurry volume given to each plant was 1 mL for most species, but as low as 0.5 mL and as high as 2 mL for some species. The cultured inoculum from the crushed nodules used the same amount of surface‐sterilized fresh nodules to start the culturing process. The noninoculated treatment received an equivalent volume of sterilized slurry or sterilized culture. Based on the success of nodulation (determined by inspection of roots of extra individuals that were not part of the main experiment), some species were reinoculated a second or a third time. Using established techniques to avoid contamination (Menge et al., 2015; Wolf et al., 2017), we placed the inoculated and uninoculated pots in separate trays, covered the surface of each pot (except where the stem protruded) with aluminum foil, and watered from below. We did not inoculate any of the plants with mycorrhizal fungi.

We fertilized plants biweekly at the top of the pots, using pipettes to add N, an N‐free Hoaglands solution (Ross, 1974), and additional P (sodium phosphate) as required by the experimental design (see below). All fertilizers were dissolved in water to facilitate their spreading throughout the rooting zone. The N fertilizer was ammonium nitrate, which was doubly labeled with ^15^N (Sigma Aldrich) for measuring N fixation. We added water via pipette at the top of each pot following each fertilization to even out the small water volume disparity across treatments.

We used 15 plant species (Table S1). Eight were rhizobial tree species, six were actinorhizal tree species, and one was the agricultural herb soybean (Table S1). The tree species we used are generally early successional or disturbance‐adapted species that grow in full or partial sun. Our initial plan was to grow all species from the first experiment in the second experiment in addition to new species, but some plants did not germinate or form a symbiosis or survive, so we present results from eight rhizobial tree species in Barnard, five rhizobial tree species at UC Davis, six actinorhizal tree species at UC Davis, and soybean at UC Davis. Different species were grown for different lengths of time, though all were less than a year (Table S2). Within each species, all plants were harvested within as short a time window as possible, and the harvest order was randomized across treatment. We harvested plants when they had grown long enough for treatment differences to appear but not so long that pot‐binding or cross‐contamination of the uninoculated plants was likely.

### 2.2 Experimental design

Our study used a factorial combination of inoculation and fertilization. We inoculated half the plants and left the other half uninoculated. Some (13% across both experiments) uninoculated plants grew nodules, but we did not include those in our analysis. For the fertilization component of the design, we used a replicated regression design, distributing our experimental units across a wide gradient with some replication within each unit, which has benefits of statistical power as well as applicability to models (Cottingham et al., 2005). For most species, there were 10 fertilization treatments: nine N fertilization levels at a low P fertilization level, along with a high P fertilization level at the highest N fertilization level. We had hoped to assess the role of P limitation in addition to the role of N limitation, but P did not limit growth in the plants grown at UC Davis (see below). For this reason, as well as the low sample size in the high P treatments, we focus less on the data from the P fertilization treatment.

Our goal for the N fertilization levels was to span a wide range of N limitation for the uninoculated plants, with multiple treatments that were N limited and multiple treatments that were not N limited. The goal of having multiple treatments that were not N limited was to determine whether N fixation shut off completely when soil N supply was sufficient, so we could test theory about N fixation strategies (Menge et al., 2009, 2015, 2023). In the present paper, it was not essential to reach levels of N sufficiency, but we explain this reasoning so the following adjustments in N levels make sense. For the first experiment, in 2016 at Barnard, we used nine N fertilization levels ranging from 0.3 to 30 g N m^−2^ year^−1^ (individual levels of 0.3, 1.5, 3.3, 6.6, 10, 15, 20, 25, 30 g N m^−2^ year^−1^), with low and high P fertilization levels of 0.34 and 15 g P m^−2^ year^−1^. (All area units are pot surface area.) The first year of the experiment in Barnard, in 2016, suggested that the highest level of N fertilization did not saturate plant demand for N, so we increased the highest N addition level. The rest of the experiment in Barnard, in 2017, used a highest level of 75 g N m^−2^ year^−1^ (levels 0.3, 3.3, 6.6, 10, 15, 20, 30, 50, 75 g N m^−2^ year^−1^) along with a lower P level of 0.17 g P m^−2^ year^−1^. In the second experiment, at UC Davis in 2018, we used turface mixed with sand, and, reasoning that turface would retain nutrients better, we used a slightly lower high N level of 60 g N m^−2^ year^−1^ (levels 0.3, 0.9, 1.5, 3.3, 6.6, 10, 20, 40, 60 g N m^−2^ year^−1^). For one species, *Morella faya*, which had low germination and initial survival, we only used six levels of N fertilization (3.3, 6.6, 10, 20, 40, 60 g N m^−2^ year^−1^). We started with three replicates for each treatment, except for *Morella faya*, which had two replicates per treatment. Final sample sizes were smaller for some species due to mortality (4% of all plants after treatments began) (Table S2).

### 2.3 Biomass harvest

We harvested plants and divided them into stems, leaves, roots, and nodules. Tissues that had previously fallen in pots (mostly leaves) were included in our biomass estimates, as were leaves previously harvested for physiological measurements (which are not shown here). We dried tissues at 65°C and measured dry masses. The majority of plants we harvested did not appear pot‐bound, but as always with seedlings grown in pots, the artificial nature of the growing medium and space should be noted.

### 2.4 N fixation

We used the ^15^N‐enriched isotope pool dilution technique to measure the percent of plant N acquired from N fixation (%N_dfa_), following the general approach of Chalk (1985) and Shearer and Kohl (1986) and the details of Menge et al. (2015) and Taylor and Menge (2018). Milled tissues were sent to the UC Davis Stable Isotope Facility to determine [N] and atom % ^15^N. Atom % ^15^N of the uninoculated, non‐nodulated plants for each species and treatment, which were enriched well over background levels (up to 8 atom %), were used as the isotopic reference values for soil N uptake. Using uninoculated plants of the same species as reference plants rather than using separate nonfixing species overcomes many of the issues with this approach (explained in more detail in Menge et al., 2015). Using enriched isotopes rather than relying on natural abundance levels overcomes many of the remaining issues (Chalk, 1985; Soper et al., 2021). We mathematically removed the effects of seed N, so %N_dfa_ is the % of newly acquired N from fixation as opposed to the % of total N from fixation.

### 2.5 Calculations and statistics

All of our allocation metrics were functions of the dry masses of the four tissue types we harvested. Aboveground biomass was calculated as the sum of leaves and stems. Belowground biomass was calculated as the sum of roots and nodules. Total biomass was calculated as the sum of aboveground and belowground biomass. Allocation of biomass to belowground versus aboveground tissues was calculated as belowground biomass divided by total biomass. Allocation to leaves versus stems was calculated as leaf biomass divided by aboveground biomass. Allocation to nodules versus roots was calculated as nodule biomass divided by belowground biomass.

To answer our questions, we used the mixed effects model function lme (Pinheiro et al., 2022) in R (R Core Team, 2022). Given the stark differences between plants grown at Barnard versus UC Davis (plants were substantially smaller and more P limited at Barnard; see Section 3) and the different environmental conditions at the two greenhouses (see Section 4), we analyzed data from each greenhouse separately. For each response variable at each greenhouse, we included a random effect of species on the intercept to account for species‐level differences.

For total biomass, our main questions were whether each symbiotic type in each greenhouse was N limited and P limited. We were less interested in the relative degrees of limitation or the relative amounts of total biomass across symbiotic types. Therefore, rather than including symbiotic type as a term in an overall model of total biomass, we analyzed the total biomass of each symbiotic type (rhizobial tree vs. actinorhizal tree vs. rhizobial herb) separately for trees grown at UC Davis, using fixed effects for N fertilization (treated throughout as a continuous variable), P fertilization, inoculation, %N_dfa_, and interactions between N fertilization and %N_dfa_, N fertilization and inoculation, P fertilization and %N_dfa_, and P fertilization and inoculation. In a separate set of analyses, we used the rate of N fixation (N fixed g C^−1^ year^−1^) rather than the percent of N derived from N fixation (%N_dfa_) as the “N fixation” driver variable. The qualitative results of these analyses with N fixed g C^−1^ year^−1^ were similar to the results from the analyses with %N_dfa_ (Note S1; Tables S3 and S4). We included the analyses with %N_dfa_ in the main text, leaving the alternate analyses to Data S1, because %N_dfa_ was the quantity we measured more directly.

Whereas we separated symbiotic types for analyses of biomass, we combined the symbiotic types for analyses about allocation and included symbiotic type as a term in the models. The reason was that one of our questions was how these allocation patterns differed across symbiotic types. Therefore, including all symbiotic types in the same model allowed us to compare the trends directly.

For allocation of total biomass to belowground versus aboveground and allocation of aboveground biomass to leaves versus stems, we used similar model structures to the one for total biomass, except that we added the natural logarithm of biomass as a covariate, and as mentioned in the previous paragraph, we included symbiotic type as a driver (at UC Davis only, given that there was only one symbiotic type at Barnard). Specifically, for the models of allocation for the UC Davis experiment, we included fixed effects of symbiotic type as well as interactions between symbiotic type and N fertilization and between symbiotic type and P fertilization (at UC Davis). We used biomass as a covariate because larger plants can have different biomass allocation than smaller plants independent of nutrient effects (McCarthy & Enquist, 2007; Poorter et al., 2012), and we wanted to control for these indirect effects. For instance, if N fertilization makes plants bigger, it might cause them to invest relatively more biomass in stems compared to leaves simply because they are bigger (and bigger plants need more mechanical support to counter gravity), whereas we wanted to isolate the effect of N fertilization for a given size.

For allocation of belowground biomass to nodules versus roots, we only used inoculated plants, so we did not include fixed effects for inoculation. The fixed main effects we used for allocation to nodules versus roots were N fertilization, P fertilization, symbiotic type (for the experiment in UC Davis), and the natural logarithm of biomass. We also used fixed interactions between N fertilization and symbiotic type for the experiment in UC Davis. Nodule biomass drives N fixation, so it did not make sense to include %N_dfa_ as a driver of nodule biomass.

## 3 RESULTS

Unless otherwise specified, results come from our statistical models (Equations (1), (2), (3), (4), (5), (6), (7), (8), (9), (10)). These are presented as an average plant's expected response to a driver variable in a scenario. For example, to illustrate the effect of N fixation on allocation, we plug in values for the other variables corresponding to a scenario, then compare the results from multiple values of N fixation. “The average inoculated plant at low N” means that we plug in a value of 1 for *I* (“inoculated”) and the lowest value of N fertilization for *N*. We then compare the model output for two separate values for %N_dfa_, such as 0% and 100%. These are not averages from inoculated plants with exactly 0% N_dfa_ and exactly 100% N_dfa_; they are the results for hypothetical average inoculated plants when we plug in 0% and 100% for %N_dfa_, as informed by all the data that were used to fit the model.

### 3.1 Total biomass

We set up our experiments in the hope that plants would be N limited at the low N fertilization levels, so we expected to find N limitation. Encouragingly, we did.

For the species grown at Barnard, all of which were rhizobial trees, the fixed effects from the mixed model were

(1) $$\begin{array}{c} \text{Barnard rhizobial tree total biomass}\;\left(\mathrm{mg}\right)\\ \;=1247+28.9*N-294*I+20.3*\%N_{\mathrm{dfa}}\\ \;+209*P+5.95*N*I+0.497*N*\%N_{\mathrm{dfa}}\\ \;+46.4*P*I+0.282*P*\%N_{\mathrm{dfa}} \end{array}$$

where *N* is N supplied as fertilizer (g N m^−2^ year^−1^), *I* is inoculated (1 if inoculated, 0 if uninoculated), %*N*_*dfa*_ is the fraction of the plant's N from fixation (%), and *P* is P supplied as fertilizer (g P m^−2^ year^−1^). Coefficients aside from the intercept that are significantly different from zero (*p* < .05) are shown in bold along with their respective variables. *p* values corresponding to each of the coefficients in Equations (1–10) are shown in Table 1.

**TABLE 1:** Significance (*p* values) of fixed effect coefficients for statistical models shown in Equations (1‐10) in the results text.

Response variable	$\beta_{N}$ *	$\beta_{I}$	$\beta_{N_{\mathrm{dfa}}}$	$\beta_{P}$	$\beta_{A}$	$\beta_{S}$	$\beta_{\ln\left(B\right)}$	$\beta_{N\times I}$	$\beta_{N\times N\mathrm{dfa}}$	$\beta_{N\times A}$	$\beta_{N\times S}$	$\beta_{P\times I}$	$\beta_{P\times N\mathrm{dfa}}$	$\beta_{P\times A}$	$\beta_{P\times S}$
Barnard rhizobial tree total biomass (mg)	**<.0001**	−.3524	**.0007**	**<.0001**				.5382	.2562			.3675	.8908		
UC Davis rhizobial tree total biomass (mg)	**<.0001**	−.4806	<**.0001**	−.5156				−.6476	<**.0001**			−.9726	−.9429		
UC Davis actinorhizal tree total biomass (mg)	**<.0001**	−**.0221**	**<.0001**	.7550				−*.0647*	**.0005**			−.3095	.3689		
UC Davis soybean total biomass (mg)	**<.0001**	.2621	**.0003**	.7206				.5705	.1668			.4393	−*.0635*		
Barnard belowground biomass (% of total)	–**<.0001**	−.2912	−**.0004**	−**.0319**			.6313	.1326	.9895			−.3804	.5563		
UC Davis belowground biomass (% of total)	–**<.0001**	−.2507	–**<.0001**	.1004	−.5905	−.1020	**.0007**	**.0276**	.4508	.4903	.7675	−.6748	−.7671	.7125	−.6717
Barnard foliar biomass (% of aboveground)	.2204	−**.0309**	**<.0001**	.4359			–**<.0001**	.3546	.4387			−.5832	−.9277		
UC Davis foliar biomass (% of aboveground)	.3659	.7126	**<.0001**	−**.0072**	.0927	−.4011	–**<.0001**	−.2320	−**.0024**	.3818	<**.0001**	.2767	.7729	.4661	−.8535
Barnard nodule biomass (% of belowground)	–**<.0001**			**.0196**			**<.0001**								
UC Davis nodule biomass (% of belowground)	–**<.0001**			.5898	−.1123	−.1859	.8672			**.0066**	.4276			−.3690	−.8777

* *p* values are shown as negative when the effect is negative. *p* values are shown in bold when they are <.05 and in italics when they are between .05 and .1. The coefficient names correspond to driver variables *N* (N supplied as fertilizer), *I* (inoculation), *N*_*dfa*_ (the fraction of the plant's N from fixation), *P* (P supplied as fertilizer), *A* (actinorhizal tree as opposed to rhizobial tree), *S* (soybean as opposed to rhizobial tree), ln(*B*) (the natural logarithm of biomass), and interactions between some pairs of these variables.

**FIGURE 1:**
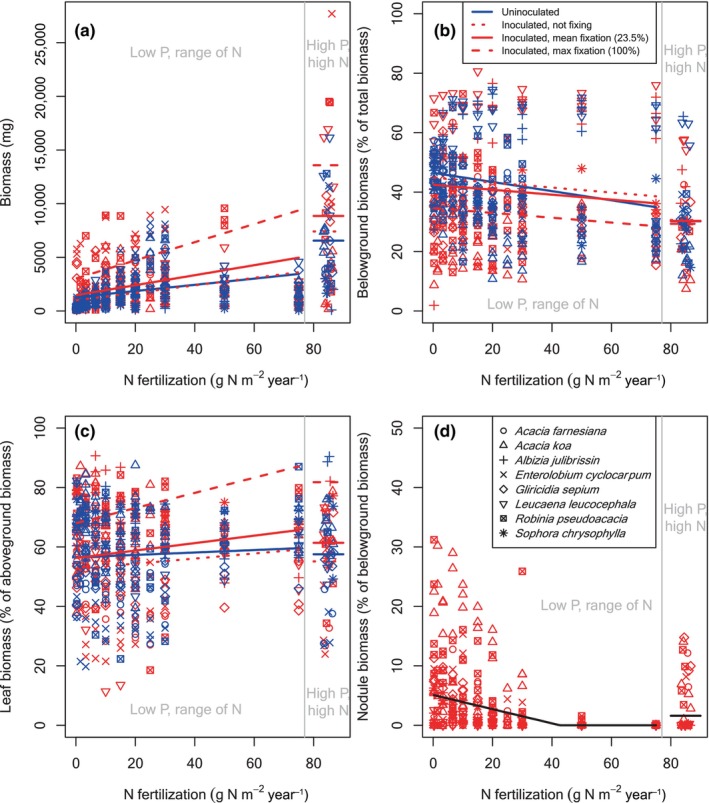
Biomass and biomass allocation across an N fertilization gradient for plants grown at Barnard. (a) Total biomass, (b) the fraction of biomass allocated belowground, (c) the fraction of aboveground biomass allocated to leaves, and (d) the fraction of belowground biomass allocated to nodules, each as a function of N fertilization. High P fertilization points, which have the same N fertilization level as the highest N fertilization points to the left of the gray line, are shown to the right of the gray line. Allocation to nodules is only shown for inoculated plants, as any uninoculated plants that grew nodules were excluded from the analysis. Legends shown in (b) and (d) apply to all panels. As the legend shows, different species are shown as different symbols. Blue symbols are uninoculated; red symbols are inoculated. Each symbol is one individual plant. Lines are the fixed effects from the mixed effects model, evaluated at the average plant biomass for each group (uninoculated vs. inoculated, low vs. high P). For uninoculated fits, both “*I*” and “*%N*_*dfa*_” are set to 0. For inoculated fits, “*I*” is set to 1, and three fits are shown, corresponding to three values of N fixation: 0 %N_dfa_, the mean %N_dfa_ within each greenhouse, and 100% N_dfa_. Blue and red colors on lines correspond to the points. The fit in (d) is shown in black instead of red to make it easier to see.

**FIGURE 2:**
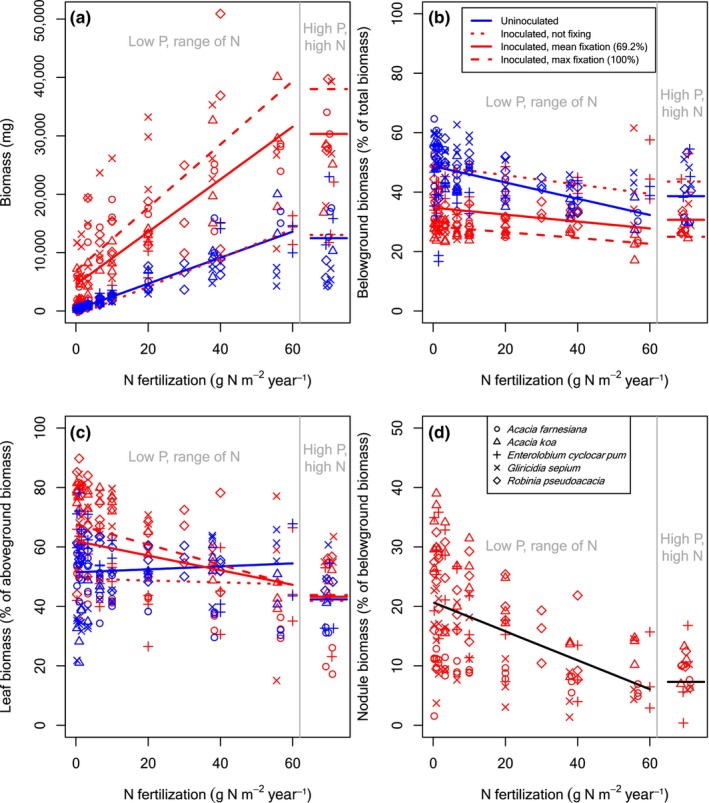
Biomass and biomass allocation for rhizobial tree seedlings across an N fertilization gradient for plants grown at UC Davis. Details as in Figure 1.

**FIGURE 3:**
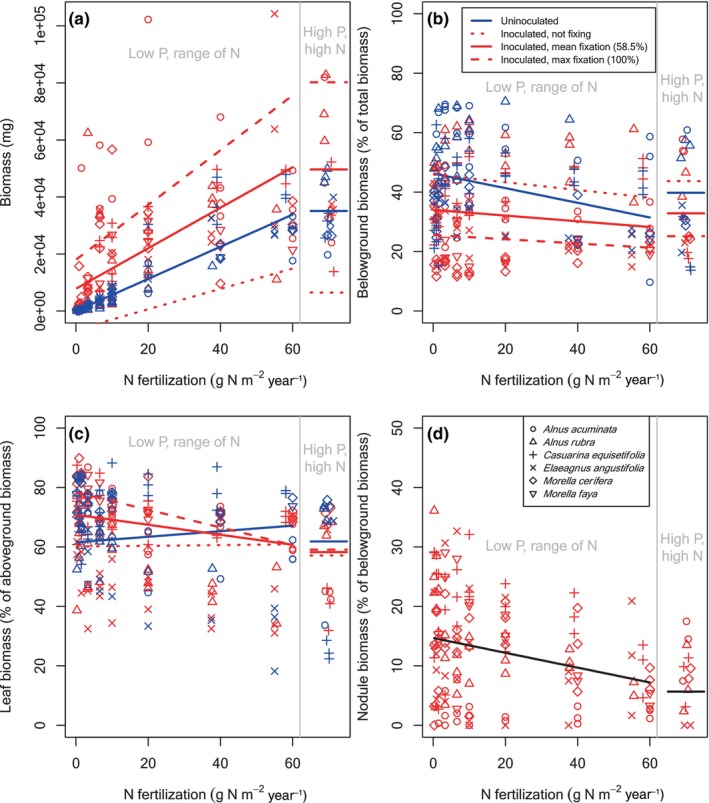
Biomass and biomass allocation for actinorhizal tree seedlings across an N fertilization gradient for plants grown at UC Davis. Details as in Figure 1.

**FIGURE 4:**
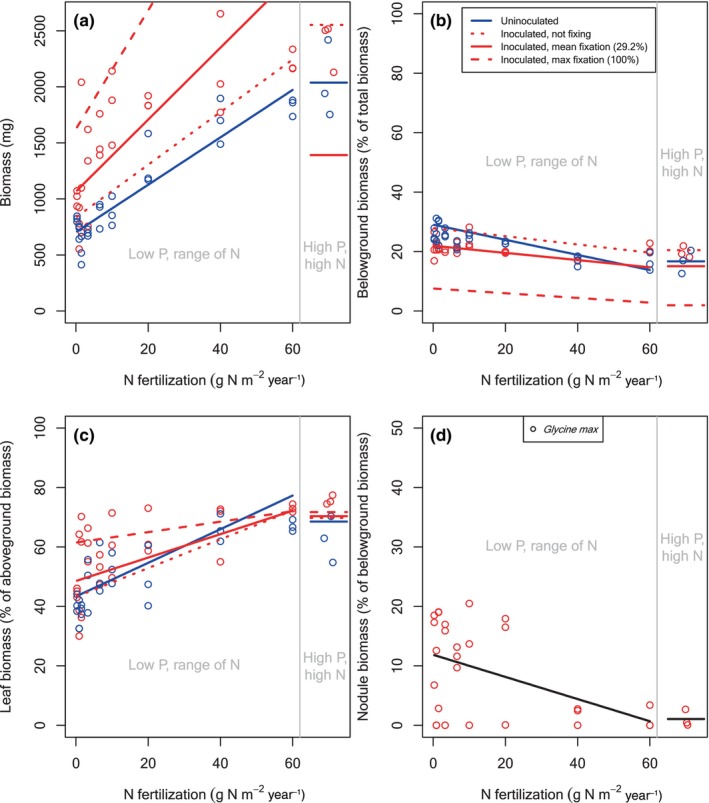
Biomass and biomass allocation for soybean plants grown across an N fertilization gradient at UC Davis. Details as in Figure 1.

N fertilization made rhizobial tree seedlings at Barnard larger—every additional g N m^−2^ year^−1^ led to 28.9 mg more biomass for uninoculated seedlings and 34.9 mg more biomass for inoculated seedlings (*p* < .0001 for both), indicating that they were N limited (blue line in Figure 1a). Given this evidence for N limitation, it is not surprising that N fixation also made seedlings larger: at low soil N supply, each percentage point of N_dfa_ led to 20.3 mg more biomass (*p* = .0007) (compare the three red lines in Figure 1a). (Note that the lines in Figures 1, 2, 3, 4 and Figures S1–S8 were calculated in the same way as described at the beginning of the Section 3.) N fixation and N fertilizer did not interact (*p* = .2562 for the interaction coefficient 0.497), meaning that N fixation led to similar increases in biomass regardless of the level of N fertilization, and conversely, N fertilization led to similar increases in biomass regardless of the amount of N fixation (compare red and blue lines in Figure 1a). Figures S1–S8 show the data for each Barnard species individually.

P fertilization, which only occurred at the highest N level, made rhizobial tree seedlings at Barnard grow larger. Each additional g P m^−2^ year^−1^ led to 209 and 256 mg more biomass for uninoculated and inoculated seedlings, respectively (*p* < .0001 for both), indicating that growth was limited by P when enough N was supplied (Figure 1a). P fertilization did not interact with inoculation (*p* = .3675 for the coefficient 46.4) or N fixation (*p* = .8908 for the coefficient 0.282), meaning that N‐fixing plants were not more or less P limited than uninoculated or nonfixing plants.

Aside from its indirect effect through N fixation, inoculation did not affect biomass for the rhizobial plants grown at Barnard. Neither the main effect of inoculation nor its interactive effects with other drivers were significantly different from zero (Table 1; see also blue vs. red dotted line in Figure 1a, which shows the average biomasses of uninoculated plants vs. inoculated plants that are not fixing N, as given by our statistical model).

For the plants grown at UC Davis, the fixed effects from the mixed model were

(2) $$\begin{array}{c} \mathrm{UC}\;\text{Davis rhizobial tree seedling total biomass}\;(\mathrm{mg})\\ \;=254+222*N-1009*I+76.6*\%N_{\mathrm{dfa}}\\ \;-74.9*P+23.9*N*I+2.95*N*\%N_{\mathrm{dfa}}\\ \;+9.10*P*I-0.241*P*\%N_{\mathrm{dfa}} \end{array}$$

(3) $$\begin{array}{c} \mathrm{UC}\;\text{Davis actinorhizal tree seedling total biomass}\;\left(\mathrm{mg}\right)\\ \;=-19.1+564*N-6408*I+244*\%N_{\mathrm{dfa}}\\ \;+83.7*P-207*N*I+6.04*N*\%N_{\mathrm{dfa}}\\ \;-650*P*I+8.78*P*\%N_{\mathrm{dfa}} \end{array}$$

(4) $$\begin{array}{c} \mathrm{UC}\;\text{Davis soybean total biomass}\;\left(\mathrm{mg}\right)\\ \;=703+21.1*N+129*I+8.63*\%N_{\mathrm{dfa}}\\ \;+4.43*P+2.33*N*I+0.295*N*\%N_{\mathrm{dfa}}\\ \;+16.4*P*I-4.40*P*\%N_{\mathrm{dfa}} \end{array}$$

As we observed at Barnard, N fertilization stimulated growth at UC Davis, as all symbiotic types at UC Davis were N limited (*p* < .0003 for uninoculated and inoculated seedlings of all symbiotic types). The magnitude at UC Davis was also much larger for the tree species types compared to Barnard. Each g N m^−2^ year^−1^ fertilizer led to 222 and 564 mg biomass for every g N m^−2^ year^−1^ added to uninoculated rhizobial (Figure 2a) and actinorhizal (Figure 3a) trees, respectively. Similarly, N fixation stimulated growth at low soil N supply more so for the tree seedlings at UC Davis than at Barnard: each additional % of fixation led to 76.6 and 244 additional mg of biomass at low soil N supply for the rhizobial and actinorhizal trees at UC Davis (*p* < .0001) (compare red line intercepts in Figures 2a and 3a). Unlike what we observed in the Barnard plants, N fixation and N fertilization interacted in the UC Davis tree seedlings (*p* < .0001 and *p* = .0005 for rhizobial and actinorhizal seedlings, respectively). Somewhat surprisingly, given that soil N and N fixation provide the same resource, the interaction was synergistic: an inoculated tree seedling with 100% N_dfa_ grew an additional 294 (rhizobial) or 604 (actinorhizal) mg with each g N m^−2^ year^−1^ of N fertilizer compared to an inoculated but nonfixing (0% N_dfa_) seedling (compare slopes of dashed vs. dotted red lines in Figures 2a and 3a). This synergy could stem from the exponential nature of seedling growth: an initial edge from fertilization could be compounded to a much greater biomass advantage even if the large majority of N comes from fixation. (We note again that the “100% N_dfa_” case is the edge case of a statistical extrapolation from plants that fixed less than 100% of their N.).

Similar to the tree species, soybean plants at UC Davis grew larger with additional N (Figure 4a). Soybean plants were smaller than tree seedlings, in part because they had less time to grow, so they grew less with each additional g N m^−2^ year^−1^ fertilizer (21.1 mg for uninoculated plants) and each % of N from fixation (8.63 mg for uninoculated plants) than the tree seedlings, but the effects were similarly significant (*p* < .0001, *p* = .0003, respectively; Table 1). Unlike the tree seedlings at UC Davis, there was no interaction between N fertilization and %N_dfa_ for soybean (*p* = .1668).

P fertilization had no effect on biomass at UC Davis for any symbiotic type (Figures 2, 3, 4). Although it was not significant, the interaction of P with N fixation makes the high P fit for soybean (Figure 4a) appear too low. The reason is that it is plotted for the average %N_dfa_ of all inoculated soybean plants, whereas the highest N, high P soybean plants had low %N_dfa_. In addition to its effects through N fixation, inoculation had a countering effect on the intercept for actinorhizal trees (*p* = .0221), but not for rhizobial trees or soybean (Table 1). Aside from its effects through N fixation, inoculation did not modify the effects of N fertilization or P fertilization (Table 1).

Given that plants were N limited in both experiments, and that both N fertilization and N fixation stimulated growth, both experiments were well suited to addressing our questions about allocation with regard to N supply via fertilization and N fixation. With regard to P supply, though, the plants grown at Barnard were P limited, but the plants grown at UC Davis were not.

### 3.2 Allocation of biomass to belowground versus aboveground tissues

Allocation to belowground versus aboveground tissues varied widely across species and treatments, ranging from ~10% to ~80% belowground for individual plants (Figures 1, 2, 3, 4 and Figures S1–S8). Species differences accounted for some of this variation (Figures 1, 2, 3, 4 and Figures S1–S8, Table 2). In Barnard, the random effect intercepts (species‐level % belowground for uninoculated plants at low soil N supply, low soil P supply, and low biomass) ranged from 29% belowground for *Acacia koa* to 66% belowground for *Leucaena leucocephala* (Table 2). In UC Davis, the range was similar, from 27% for *Morella faya* to 56% for *Alnus rubra* (Table 2). Across all species and treatments, the means of all individual plants were 39% belowground at Barnard and 36% belowground at UC Davis.

**TABLE 2:** Random intercepts for statistical models.

Species^a^	Barnard			UC Davis		
	Belowground (% of total)	Foliar (% of aboveground)	Nodule (% of belowground)	Belowground (% of total)	Foliar (% of aboveground)	Nodule (% of belowground)
*Acacia farnesiana* ^R^	44.1	63.7	−8.52	37.7	57.8	13.4
*Acacia koa* ^R^	29.4	83.3	−1.67	34.2	69.7	26.3
*Albizia julibrissin* ^R^	60.4	83.8	−6.61			
*Alnus acuminata* ^A^				46.8	70.1	10.9
*Alnus rubra* ^A^				55.8	59.2	22.3
*Casuarina equisetifolia* ^A^				41.7	76.4	25.3
*Elaeagnus angustifolia* ^A^				27.9	49.6	19.5
*Enterolobium cyclocarpum* ^R^	38.9	58.5	−9.40	34.5	70.7	22.4
*Gliricidia sepium* ^R^	39.4	67.4	−5.97	43.0	69.6	16.2
*Glycine max* (soybean)^H^				38.1	68.3	20.2
*Leucaena leucocephala* ^R^	66.0	76.5	−9.00			
*Morella cerifera* ^A^				29.9	77.0	19.8
*Morella faya* ^A^				26.5	77.6	23.3
*Robinia pseudoacacia* ^R^	39.9	79.6	−4.49	41.0	73.8	22.6
*Sophora chrysophylla* ^R^	40.0	84.5	−6.28			

*Note*: Five species were grown in both greenhouses, whereas 10 were only grown in one (Barnard or UC Davis).

^a^ Superscript codes: R is rhizobial tree, A is actinorhizal tree, H is rhizobial herb.

Given that plants in both greenhouses were N limited, we hypothesized (H1a) that N fertilization and N fixation would induce plants to allocate relatively less biomass belowground, which would appear as negative coefficients for the “N” and “%N_dfa_” terms in the mixed models for belowground biomass as a % of total biomass. The fixed effects from the mixed model were

(5) $$\begin{array}{c} \text{Barnard belowground biomass}\;\left(\%\text{of total}\right)\\ \;=44.8-0.154*N-1.61*I-0.104*\%N_{\mathrm{dfa}}\\ \;-0.345*P+0.258*\ln\left(\text{Biomass}\right)+0.0702*N*I\\ \;+0.00003*N*\%N_{\mathrm{dfa}}-0.218\\ \;*P*I+0.00591*P*\%N_{\mathrm{dfa}} \end{array}$$

(6) $$\begin{array}{c} \mathrm{UC}\;\text{Davis plant belowground biomass}\;\left(\%\text{of total}\right)\\ \;=38.1-0.273*N-1.70*I-0.203*\%N_{\mathrm{dfa}}\\ \;+0.261*P+1.42*\ln\left(\text{Biomass}\right)-18.8*S\\ -3.20*A+0.116*N*I+0.00059*N*\%N_{\mathrm{dfa}}\\ \;+0.0189*N*S+0.0267*N*A-0.115*P*I\\ \;-0.00118*P*\%N_{\mathrm{dfa}}-0.136*P*S+0.0677*P*A \end{array}$$

where *S* indicates soybean (1 if soybean, 0 if not) and *A* indicates actinorhizal tree (1 if actinorhizal tree, 0 if not). As we had hypothesized (H1a), plants in both locations allocated less biomass belowground when they had more N, either from fertilization or from N fixation. At Barnard, the average uninoculated plant allocated 46% belowground at our lowest N fertilization level, compared to 35% at the highest N level (Figure 1b, blue line). The effect of N fixation was similar to the effect of N fertilization. As explained at the beginning of the Section 3, we illustrate this by comparing the hypothetical cases of %N_dfa_ = 0 versus 100% in Equation (5). The average inoculated plant at low N at Barnard allocated 45% belowground at 0% N_dfa_, compared to 35% belowground at 100% N_dfa_ (compare red lines in Figure 1b). N fixation did not interact with soil N supply for the Barnard plants, so at high N, the average inoculated rhizobial plant allocated 39% belowground if it was not fixing, compared to 29% belowground at 100% N_dfa_ (compare red lines in Figure 1b). Our hypothesis about a negligible effect of inoculation aside from its effect on N fixation (H1b) was supported for the Barnard plants: neither the main effect of inoculation nor its interaction effects were significant.

Just like the N effects on biomass, the N effects on aboveground versus belowground biomass allocation were similar in direction but more drastic in magnitude at UC Davis compared to Barnard. At UC Davis, the average uninoculated rhizobial tree seedling allocated 49% (46% for actinorhizals) belowground at the lowest N level and 32% (31% for actinorhizals) at the highest N level (Figures 2b and 3b). Fixation had at least as large an effect as N fertilization. An average inoculated rhizobial tree seedling at low N allocated 49% (46% for actinorhizals) belowground if it was fixing 0% of its N compared to 28% (26% for actinorhizals) if it was fixing 100% of its N (compare left side of red lines, Figures 2b and 3b). At the highest N level, an average inoculated rhizobial tree seedling allocated 39% (38% for actinorhizals) belowground if it was fixing 0% of its N, whereas it allocated 23% (21%) belowground when it was fixing 100% of its N (compare right side of red lines, Figures 2b and 3b). Furthermore, N fixation levels were higher at UC Davis: an average of 69 %N_dfa_ for rhizobial tree seedlings at UC Davis compared to 24% at Barnard (and 59% for actinorhizal tree seedlings at UC Davis). Therefore, the large effects at UC Davis were even stronger than they appear in the coefficients: the effects on belowground allocation were at least as large per unit %N_dfa_, but their realized effects were even larger because the plants were fixing more N. Unlike in the plants grown at Barnard, there was a significant interaction term between inoculation and N supply for the plants grown at UC Davis, indicating that inoculation had an effect apart from N fixation itself (Equation 6). Whereas an average uninoculated plant and an average inoculated but non‐fixing (0% N_dfa_) plant had similar allocation to belowground tissues at low N supply, their allocation belowground diverged at higher N supply (compare the blue vs. dotted red lines in Figures 2b and 3b). Therefore, H1b had only partial support from the plants grown at UC Davis.

Though not significant, soybean tended to allocate less biomass belowground than rhizobial tree seedlings. The effects of N on allocation were similar in soybean and the other plants, though. At UC Davis, the average uninoculated soybean seedling allocated 29% belowground at the lowest N level and 14% at the highest N level (Figure 4b). Fixation had at least as large an effect as N fertilization. The average inoculated soybean seedling at low N allocated 28% belowground if it was fixing 0% of its N, compared to 8% if it was fixing 100% of its N (compare left side of dashed vs. dotted red lines, Figure 4b). At the highest N level, the average inoculated soybean seedling allocated 20% belowground if it was not fixing, whereas it allocated 3% belowground when it was fixing 100% of its N (compare right side of dashed vs. dotted red lines, Figure 4b).

### 3.3 Allocation of aboveground biomass to leaves versus stems

The fraction of aboveground biomass allocated to leaves ranged widely, from ~10% to ~90% for individual plants (Figures 1, 2, 3, 4). As was the case with allocation to belowground biomass, there was substantial variation across species. At Barnard, the random effect intercepts ranged from 59% leaves for *Enterolobium* to 85% leaves for *Sophora* (Table 2). At UC Davis, the random effects intercepts ranged from 50% leaves (*Elaeagnus*) to 78% leaves (*Morella faya*) (Table 2). Across all species and treatments, the average allocation to aboveground tissue was 59%.

We did not have a clear hypothesis for how N fertilization and N fixation would affect allocation to stems versus leaves, as H2a, b, and c were mutually exclusive alternatives. Accordingly, the results were nuanced. The fixed effects from the mixed models were

(7) $$\begin{array}{c} \text{Barnard foliar biomass}\;\left(\%\text{of aboveground}\right)\\ \;=74.7+0.0411*N-3.38*I+0.153*\%N_{\mathrm{dfa}}\\ \;+0.128*P-2.64*\ln\left(\text{Biomass}\right)+0.0442*N*I\\ \;+0.00170*N*\%N_{\mathrm{dfa}}-0.140*P*I\\ \;-0.00093*P*\%N_{\mathrm{dfa}} \end{array}$$

(8) $$\begin{array}{c} \mathrm{UC}\;\text{Davis foliar biomass}\;\left(\%\text{of aboveground}\right)\\ \;=68.3+0.0497*N+0.703*I+0.183*\%N_{\mathrm{dfa}}\\ \;-0.552*P-2.25*\ln\left(\text{Biomass}\right)-9.45*S\\ \;+11.2*A-0.0813*N*I-0.00309*N*\%N_{\mathrm{dfa}}\\ \;+0.516*N*S+0.0438*N*A+0.386*P*I\\ \;+0.00148*P*\%N_{\mathrm{dfa}}+0.0766*P*S+0.173*P*A \end{array}$$

As expected, larger plants invested relatively more in stems as opposed to leaves (*p* < .0001 for both locations for the effect of the natural log of biomass; Table 1). As we mentioned in the biomass section, N fertilization and N fixation both made plants bigger, so there was an indirect effect whereby N supply (via fertilization and fixation) caused plants to invest relatively more in stems because it made them bigger. However, this indirect effect was countered by a direct effect: for a given size, N fixation (*p* < .0001 for both Barnard and UC Davis) stimulated plants to invest more in leaves. N fertilization had a similar effect in soybean at UC Davis. These combined effects are visible (Figures 1, 2, 3, 4). The fit for uninoculated plants (blue line) in Figure 3c, for instance, rises more than seems warranted by the points. This occurs because the fit is plotted for an average‐sized actinorhizal seedling, whereas in reality, the plants at low N fertilization were small (and thus had higher investment in leaves than indicated by the fit) and the plants at high N fertilization were large (and thus had lower investment in leaves than indicated by the fit). In UC Davis, N fertilization had the opposite effect at low versus high N fixation: it caused more investment in leaves at low N fertilization but had no effect at high fertilization (Figures 2, 3, 4). Overall, there was mixed support for H2a (an increase in allocation to leaves vs. stems with more N) versus H2b (no change) versus H2c (decrease).

### 3.4 Allocation of belowground biomass to nodules versus roots

Investment in nodules also ranged widely across plants, from 0% to nearly 40% of belowground biomass (Figures 1, 2, 3, 4). Species varied widely; across species, the average allocation to nodules in inoculated plants was 4.1% in Barnard, compared to 13.6% in UC Davis.

We had hypothesized that N fertilization would reduce investment in nodules (H3). As the fixed effects equations show, our data supported H3:

(9) $$\begin{array}{c} \text{Barnard nodule biomass}\;\left(\%\text{of belowground}\right)\\ \;=-6.49-0.120*N+1.68*\ln\left(\text{Biomass}\right)\\ \;+0.189*P \end{array}$$

(10) $$\begin{array}{c} \mathrm{UC}\;\text{Davis nodule biomass}\;\left(\%\text{of belowground}\right)\\ \;=20.2-0.243*N+0.0562*\ln\left(\text{Biomass}\right)\\ \;+0.0785*P-8.72*S-5.96*A+0.0571*N*S\\ \;+0.119*N*A \end{array}$$

For the plants grown at Barnard, an average‐sized individual at our low P level allocated about 5% of its belowground biomass to nodules at the lowest N level, which dropped to 0% by the high levels of N fertilization (Figure 1d). Larger plants allocated proportionately more belowground biomass to nodules. Fertilization with P stimulated allocation to nodules (Table 1, Figure 1d).

At UC Davis, an average‐sized rhizobial tree seedling at our low P and lowest N levels allocated approximately 21% of its belowground biomass to nodules (Figure 2d), compared to approximately 15% for actinorhizal tree seedlings (Figure 3d) and 12% for soybean plants (Figure 4d). At our highest N levels, allocation to nodules dropped to 6% of belowground biomass for rhizobials (Figure 2d), 7% for actinorhizal trees (Figure 3d), and <1% for soybean (Figure 4d). Unlike at Barnard, larger plants grown at UC Davis did not allocate more to nodules, and there was no effect of P fertilization (Table 1).

## 4 DISCUSSION

Despite wide variation in allocation of biomass to different organs across our species, and despite variable growing conditions in the two experiments, we found a number of consistent patterns. As we had hypothesized (H1a), and as many others had found with non‐fixing species (Brenchley, 1916; Chapin III, 1980; Ingestad & Agren, 1991; McCarthy & Enquist, 2007; Poorter et al., 2012; Poorter & Nagel, 2000), fertilizing uninoculated seedlings with N led to relatively less allocation of biomass to belowground tissues. The rest of our hypothesis H1 was also correct: N fixation also led to relatively less allocation of biomass belowground, with a similar overall effect size as N fertilization (as observed by Ingestad, 1980; Sellstedt, 1986; Sellstedt & Huss‐Danell, 1986), and inoculation had negligible effects aside from allowing N fixation. We had multiple competing hypotheses for allocation of aboveground biomass to leaves versus stems (H2a, b, and c). Accordingly, our results for leaves versus stems were variable. The clearest trend was that N fixation led to more allocation to leaves at low soil N supply, but the more nuanced results differed across our two experiments. As we had hypothesized (H3), and as many (e.g., Dovrat et al., 2018, 2020; Ingestad, 1980; Markham & Zekveld, 2007; McCulloch & Porder, 2021; Menge et al., 2015; Taylor & Menge, 2018) but not all (Arnone III & Gordon, 1990; Binkley et al., 1994) other studies had observed, fertilization decreased allocation to root nodules.

One major advantage of our work compared to past work studying the effects of N fixation on biomass allocation is our ability to compare across different groups of N‐fixing species. Our study examined 15 species overall, including 12 species from three different plant types (rhizobial trees, actinorhizal trees, and a rhizobial herb) in the second experiment. Except for a few nuances, rhizobial and actinorhizal trees had similar average patterns of biomass allocation, though there was substantial variability within each group. Therefore, the differences observed in the actinorhizal tree *Alnus* (Arnone III & Gordon, 1990; Ingestad, 1980; Markham & Zekveld, 2007; Sellstedt, 1986; Sellstedt & Huss‐Danell, 1986) versus three Mediterranean shrubs (Dovrat et al., 2020) versus the tropical rhizobial tree *Pentaclethra macroloba* (Taylor & Menge, 2021) seem not to hold generally across their plant types. We speculate that their common ecological role as woody N‐fixing plants helps explain their similar average patterns of allocation, and we also note that within‐family variation was high, as is common for many traits (Anderegg et al., 2018). Soybean was different than the tree species: it allocated proportionately more aboveground and less to nodules, and it consistently allocated more to leaves rather than stems with increasing N fertilization. The fact that herbs and trees have different allocation patterns is not that surprising, particularly with respect to leaves versus stems. However, there are additional reasons beyond being an herb that soybean might differ. As an agricultural species, soybean has been selected for fruit production in typically nutrient‐rich (fertilized) habitats, which likely means it has been selected for more allocation aboveground.

We designed our study to tease apart the roles of inoculation versus N fixation, given past findings that the symbiotic association with bacteria can have effects beyond supplying N. For example, Wolf et al. (2017) found that inoculation with symbiotic bacteria led to higher concentrations of N in plant tissues beyond what could be explained by the amount of N they fixed, and Dovrat et al. (2020) found that inoculating N‐sufficient plants caused them to allocate relatively less biomass belowground. The mechanisms underlying such effects are unclear, though it is known that the symbiotic interaction involves a series of chemical signals, both during the onset of the symbiotic interaction and once the bacteria are inside the plant (Franche et al., 2009; Garg & Manchanda, 2009). This signaling has myriad effects on plant cellular function and gene expression (Franche et al., 2009; Garg & Manchanda, 2009), so it seems plausible that it could affect biomass allocation. However, in the present study, unlike these other studies, the effects of inoculation acted primarily through N fixation. With some nuances, allocation was similar for inoculated but nonfixing versus uninoculated plants, after correcting for size (compare dotted red and solid blue lines in Figures 1, 2, 3, 4). As we explained at the beginning of the Section 3, these conclusions come from statistical fits across the full range of N fixation rather than from isolated inoculated but nonfixing individuals. Our conclusion from this finding is that any effects of inoculation beyond N fixation, such as those observed by Wolf et al. (2017) and Dovrat et al. (2020), are inconsistent across species, conditions, or response variables.

In both experiments and across all symbiotic types, plants that fixed more N allocated substantially less belowground (compare the three red lines in Figures 1, 2, 3, 4). Despite this similar overall trend, we found some stark differences in allocation between the plants in our two experiments, largely due to differences in the amount of N fixed. In the Barnard greenhouse, where the average plant fixed only 24% of its N, allocation belowground was nearly identical for the average inoculated versus the average uninoculated plant (compare solid red and blue lines in Figure 1b). By contrast, in the UC Davis greenhouse, where the average tree seedling fixed more of its N (69% for rhizobial trees, 59% for actinorhizal trees), the average inoculated plant allocated much less biomass belowground than the average uninoculated plant, even after correcting for plant size (compare red and blue points and lines in Figures 2 and 3b). Soybean, which we only grew in UC Davis, was more similar to the rhizobial trees in Barnard, with an average of 29% N_dfa_ and similar allocation patterns for the average inoculated versus uninoculated plants. We suspect that the differences in N fixation in the two greenhouses stem from different resource availability. Some of the important environmental conditions that determine rates of N fixation are the availability of resources such as light (Myster, 2006; Schmidt et al., 2023; Taylor & Menge, 2018) or phosphorus (Batterman et al., 2013; Crews, 1993; Zheng et al., 2019), both of which likely differed between the experiments. Phosphorus limited plant growth at the highest N level in Barnard (Figure 1a) but not in UC Davis (Figures 2, 3, 4), and we suspect that light availability was higher at UC Davis as well due to its geographical location as well as the fewer number of light‐blocking buildings nearby. Greater limitation by phosphorus and light would be consistent with lower allocation to nodules across the range of soil N supply in Barnard (Figure 1d compared to Figures 2, 3, 4).

A key implication of our work is that knowing whether a species is capable of N fixation is not sufficient to determine its biomass allocation; N fixation activity is much more important. This is unfortunate. There are increasingly comprehensive lists of which taxa are capable of forming symbioses (Afkhami et al., 2018; Huss‐Danell, 1997; Sprent, 2009; Werner et al., 2014), whereas it is far harder to determine N fixation activity (Soper et al., 2021). Our evolving understanding suggests that, although there are differences in N fixation rates across different taxa (Wurzburger & Hedin, 2016), there are also differences across environmental conditions (Batterman et al., 2013; Crews, 1993; Menge et al., 2015; Myster, 2006; Schmidt et al., 2023; Taylor & Menge, 2018; Zheng et al., 2019), suggesting that identical plants in different conditions might fix N, and thus allocate biomass, differently. In addition to our Barnard versus UC Davis comparison, we also saw this trend within each experiment: much of the variation in biomass allocation within a species and treatment corresponded to variation in N fixation activity.

Our results have potential implications for competition and for carbon storage at community and ecosystem scales. Fifteen species grown under a variety of conditions suggested that N fixation leads to relatively less allocation belowground, and in N‐poor conditions, relatively more allocation to leaves than stems. At the community level, these results indicate that N fixation, analogous to high soil N supply (Dybzinski et al., 2011), intensifies aboveground competition for light. At the ecosystem scale, these patterns suggest that N fixation leads to more allocation to tissues with shorter lifespans and faster decomposition. This is consistent with the well‐known effects of N fixation (or being an N fixer) on tissue N content (Adams et al., 2016; Bytnerowicz et al., 2023; Fyllas et al., 2009; Wolf et al., 2017), which can also enhance decomposition rates, particularly for low lignin litter (Cusack et al., 2009; Melillo et al., 1982; Perakis et al., 2012). However, there are a number of caveats for these extrapolations. The present study focused on seedlings grown in pots, whereas much light competition and carbon storage are driven by mature trees in the field (Pan et al., 2011), which might have different allocation. Although we selected 15 species from both major N‐fixing symbiotic types (rhizobial and actinorhizal) and across multiple biomes, these species are still a small fraction of the N‐fixing species in the world (Afkhami et al., 2018; Werner et al., 2014). Finally, although we focused mostly on the average trends in our data, it is also noteworthy that the variation around the trends was substantial, indicating that many other factors affect biomass allocation.

## AUTHOR CONTRIBUTIONS

**Duncan N. L. Menge:** Conceptualization (equal); data curation (lead); formal analysis (lead); funding acquisition (lead); investigation (supporting); methodology (equal); project administration (lead); resources (equal); software (lead); supervision (lead); visualization (lead); writing – original draft (lead); writing – review and editing (lead). **Amelia A. Wolf:** Conceptualization (equal); funding acquisition (supporting); investigation (lead); methodology (equal); project administration (supporting); writing – review and editing (supporting). **Jennifer L. Funk:** Conceptualization (equal); funding acquisition (supporting); investigation (supporting); project administration (supporting); writing – review and editing (supporting). **Steven S. Perakis:** Conceptualization (equal); funding acquisition (supporting); project administration (supporting); writing – review and editing (supporting). **K. A. Carreras Pereira:** Investigation (supporting); writing – review and editing (supporting).

## FUNDING INFORMATION

This work was supported by the National Science Foundation under grant nos. DEB‐1457444, DEB‐1457650, and IOS‐2129542.

## CONFLICT OF INTEREST STATEMENT

The authors declare and certify that they possess no conflict of interest in the materials, subject matter, methods, and so forth contained herein.

## Supporting information


Data S1.

## Data Availability

Data and code associated with this manuscript are available from the Dryad data repository at doi:10.5061/dryad.cnp5hqc9s.

## References

[ece370309-bib-0001] Adams, M. A. , Turnbull, T. L. , Sprent, J. I. , & Buchmann, N. (2016). Legumes are different: Leaf nitrogen, photosynthesis, and water use efficiency. Proceedings of the National Academy of Sciences of the United States of America, 113, 4098–4103. 27035971 10.1073/pnas.1523936113 PMC4839396

[ece370309-bib-0002] Afkhami, M. E. , Mahler, D. L. , Burns, J. H. , Weber, M. G. , Wojciechowski, M. F. , Sprent, J. , & Strauss, S. Y. (2018). Symbioses with nitrogen‐fixing bacteria: Nodulation and phylogenetic data across legume genera. Ecology, 99, 502. 29226306 10.1002/ecy.2110

[ece370309-bib-0003] Anderegg, L. D. L. , Berner, L. T. , Badgley, G. , Sethi, M. L. , Law, B. E. , & HilleRisLambers, J. (2018). Within‐species patterns challenge our understanding of the leaf economics spectrum. Ecology Letters, 21, 734–744. 29569818 10.1111/ele.12945

[ece370309-bib-0004] Arnone, J. A., III , & Gordon, J. C. (1990). Effect of nodulation, nitrogen fixation and CO_2_ enrichment on the physiology, growth and dry mass allocation of seedlings of *Alnus rubra* bong. New Phytologist, 116, 55–66.

[ece370309-bib-0005] Batterman, S. A. , Wurzburger, N. , & Hedin, L. O. (2013). Nitrogen and phosphorus interact to control tropical symbiotic N_2_ fixation: A test in *Inga punctata* . Journal of Ecology, 101, 1400–1408.

[ece370309-bib-0006] Bazzaz, F. A. , & Grace, J. (1997). Plant resource allocation. Academic Press.

[ece370309-bib-0007] Binkley, D. , Cromack, K., Jr. , & Baker, D. D. (1994). Nitrogen fixation by red alder: Biology, rates, and controls. In D. Hibbs , D. DeBell , & R. Tarrant (Eds.), The biology and Management of red Alder (pp. 57–72). Oregon State University Press.

[ece370309-bib-0008] Bloom, A. J. , Chapin, F. S., III , & Mooney, H. A. (1985). Resource limitation in plants‐an economic analogy. Annual Review of Ecology and Systematics, 16, 363–392.

[ece370309-bib-0009] Brenchley, W. E. (1916). The effect of the concentration of the nutrient solution on the growth of barley and wheat in water cultures. Annals of Botany, 30, 77–91.

[ece370309-bib-0010] Bytnerowicz, T. A. , Funk, J. L. , Menge, D. N. L. , Perakis, S. S. , & Wolf, A. A. (2023). Leaf nitrogen affects photosynthesis and water use efficiency similarly in nitrogen‐fixing and non‐fixing trees. Journal of Ecology, 111, 2457–2471.

[ece370309-bib-0011] Carreras Pereira, K. A. , Wolf, A. A. , Kou‐Giesbrecht, S. , Akana, P. R. , Funk, J. L. , & Menge, D. N. L. (2023). Allometric relationships for eight species of 4–5 year old nitrogen‐fixing and non‐fixing trees. PLoS One, 18, e0289679. 37603572 10.1371/journal.pone.0289679 PMC10441808

[ece370309-bib-0012] Chalk, P. M. (1985). Estimation of N_2_ fixation by isotope dilution: An appraisal of techniques involving ^15^N enrichment and their application. Soil Biology and Biochemistry, 17, 389–410.

[ece370309-bib-0013] Chapin, F. S., III . (1980). The mineral nutrition of wild plants. Annual Review of Ecology and Systematics, 11, 233–260.

[ece370309-bib-0014] Cottingham, K. L. , Lennon, J. T. , & Brown, B. L. (2005). Knowing when to draw the line: Designing more informative ecological experiments. Frontiers of Ecology and the Environment, 3, 145–152.

[ece370309-bib-0015] Crews, T. E. (1993). Phosphorus regulation of nitrogen fixation in a traditional Mexican agroecosystem. Biogeochemistry, 21, 141–166.

[ece370309-bib-0016] Cusack, D. F. , Chou, W. W. , Yang, W. H. , Harmon, M. E. , Silver, W. L. , & Lidet Team . (2009). Controls on long‐term root and leaf litter decomposition in neotropical forests. Global Change Biology, 15, 1339–1355.

[ece370309-bib-0017] Dovrat, G. , Bakhshian, H. , Masci, T. , & Sheffer, E. (2020). The nitrogen economic spectrum of legume stoichiometry and fixation strategy. New Phytologist, 227, 365–375. 32175592 10.1111/nph.16543

[ece370309-bib-0018] Dovrat, G. , Masci, T. , Bakhshian, H. , Gati, E. M. , Golan, S. , & Sheffer, E. (2018). Drought‐adapted plants dramatically downregulate dinitrogen fixation: Evidences from Mediterranean legume shrubs. Journal of Ecology, 106, 1534–1544.

[ece370309-bib-0019] Dybzinski, R. , Farrior, C. , Wolf, A. , Reich, P. B. , & Pacala, S. W. (2011). Evolutionarily stable strategy carbon allocation to foliage, wood, and fine roots in trees competing for light and nitrogen: An analytically tractable, individual‐based model and quantitative comparisons to data. The American Naturalist, 177, 153–166. 10.1086/657992 21460552

[ece370309-bib-0020] Franche, C. , Lindström, K. , & Elmerich, C. (2009). Nitrogen‐fixing bacteria associated with leguminous and non‐leguminous plants. Plant and Soil, 321, 35–59.

[ece370309-bib-0021] Friend, A. D. , Lucht, W. , Rademacher, T. T. , Keribin, R. , Betts, R. , Cadule, P. , Ciais, P. , Clark, D. B. , Dankers, R. , Falloon, P. D. , & Ito, A. (2014). Carbon residence time dominates uncertainty in terrestrial vegetation responses to future climate and atmospheric CO_2_ . Proceedings of the National Academy of Sciences of the United States of America, 111, 3280–3285. 24344265 10.1073/pnas.1222477110 PMC3948236

[ece370309-bib-0022] Fyllas, N. M. , Patiño, S. , Baker, T. R. , Bielefeld Nardoto, G. , Martinelli, L. A. , Quesada, C. A. , Paiva, R. , Schwarz, M. , Horna, V. , Mercado, L. M. , & Santos, A. (2009). Basin‐wide variations in foliar properties of Amazonian forest: Phylogeny, soils and climate. Biogeosciences, 6, 2677–2708.

[ece370309-bib-0023] Garg, N. , & Manchanda, G. (2009). Symbiotic nitrogen fixation in legume nodules: Process and signalling: A review. Agronomy for Sustainable Development, 27, 59–68.

[ece370309-bib-0024] Gutschick, V. P. (1981). Evolved strategies in nitrogen acquisition by plants. American Naturalist, 118, 607–637.

[ece370309-bib-0025] Hedin, L. O. , Brookshire, E. J. , Menge, D. N. L. , & Barron, A. R. (2009). The nitrogen paradox in tropical forest ecosystems. Annual Review of Ecology, Evolution, and Systematics, 40, 613–635.

[ece370309-bib-0026] Hermans, C. , Hammond, J. P. , White, P. J. , & Verbruggen, N. (2006). How do plants respond to nutrient shortage by biomass allocation? Trends in Plant Science, 11, 610–617. 17092760 10.1016/j.tplants.2006.10.007

[ece370309-bib-0027] Huss‐Danell, K. (1997). Tansley review No. 93. Actinorhizal symbioses and their N_2_ fixation. New Phytologist, 136, 375–405. 33863007 10.1046/j.1469-8137.1997.00755.x

[ece370309-bib-0028] Ingestad, T. (1980). Growth, nutrition, and nitrogen fixation in grey alder at varied rate of nitrogen addition. Physiologia Plantarum, 50, 353–364.

[ece370309-bib-0029] Ingestad, T. , & Agren, G. I. (1991). The influence of plant nutrition on biomass allocation. Ecological Applications, 1, 168–174. 27755663 10.2307/1941809

[ece370309-bib-0030] Iwasa, Y. (2000). Dynamic optimization of plant growth. Evolutionary Ecology Research, 2, 437–455.

[ece370309-bib-0031] LeBauer, D. S. , & Treseder, K. K. (2008). Nitrogen limitation of net primary productivity in terrestrial ecosystems is globally distributed. Ecology, 89, 371–379. 18409427 10.1890/06-2057.1

[ece370309-bib-0032] Markham, J. H. , & Zekveld, C. (2007). Nitrogen fixation makes biomass allocation to roots independent of soil nitrogen supply. Botany, 85, 787–793.

[ece370309-bib-0033] Maximov, N. A. , & Yapp, R. H. (1929). The plant in relation to water: A study of the physiological basis of drought resistance. Allen and Unwin.

[ece370309-bib-0034] McCarthy, M. C. , & Enquist, B. J. (2007). Consistency between an allometric approach and optimal partitioning theory in global patterns of plant biomass allocation. Functional Ecology, 21, 713–720.

[ece370309-bib-0035] McCulloch, L. A. , & Porder, S. (2021). Light fuels while nitrogen suppresses symbiotic nitrogen fixation hotspots in neotropical canopy gap seedlings. New Phytologist, 231, 1734–1745. 34058025 10.1111/nph.17519

[ece370309-bib-0036] Melillo, J. M. , Aber, J. D. , & Muratore, J. F. (1982). Nitrogen and lignin control of hardwood leaf litter decomposition dynamics. Ecology, 63, 621–626.

[ece370309-bib-0037] Menge, D. N. L. , Batterman, S. A. , Liao, W. , Taylor, B. N. , Lichstein, J. W. , & Ángeles‐Perez, G. (2017). Nitrogen‐fixing tree abundance in higher‐latitude North America is not constrained by diversity. Ecology Letters, 20, 842–851. 28512925 10.1111/ele.12778

[ece370309-bib-0038] Menge, D. N. L. , Levin, S. A. , & Hedin, L. O. (2009). Facultative versus obligate nitrogen fixation strategies and their ecosystem consequences. American Naturalist, 174, 465–477. 10.1086/605377 19694561

[ece370309-bib-0039] Menge, D. N. L. , Wolf, A. A. , & Funk, J. L. (2015). Diversity of nitrogen fixation strategies in Mediterranean legumes. Nature Plants, 1, 1–5. 10.1038/nplants.2015.64 27250004

[ece370309-bib-0040] Menge, D. N. L. , Wolf, A. A. , Funk, J. L. , Perakis, S. S. , Akana, P. R. , Arkebauer, R. , Bytnerowicz, T. A. , Carreras Pereira, K. A. , Huddell, A. M. , Kou‐Giesbrecht, S. , & Ortiz, S. K. (2023). Tree symbioses sustain nitrogen fixation despite excess nitrogen supply. Ecological Monographs, 93, e1562.

[ece370309-bib-0041] Myster, R. W. (2006). Light and nutrient effects on growth and allocation of *Inga vera* (Leguminosae), a successional tree of Puerto Rico. Canadian Journal of Forest Research, 36, 1121–1128.

[ece370309-bib-0042] Pan, Y. , Birdsey, R. A. , Fang, J. , Houghton, R. , Kauppi, P. E. , Kurz, W. A. , Phillips, O. L. , Shvidenko, A. , Lewis, S. L. , Canadell, J. G. , & Ciais, P. (2011). A large and persistent carbon sink in the world's forests. Science, 333, 988–993. 21764754 10.1126/science.1201609

[ece370309-bib-0043] Peoples, M. B. , Giller, K. E. , Jensen, E. S. , & Herridge, D. F. (2021). Quantifying country‐for to‐global scale nitrogen fixation for grain legumes: I. Reliance of nitrogen fixation of soybean, groundnut, and pulses. Plant and Soil, 469, 1–14.

[ece370309-bib-0044] Perakis, S. S. , Matkins, J. J. , & Hibbs, D. E. (2012). Interactions of tissue and fertilizer nitrogen on decomposition dynamics of lignin‐rich conifer litter. Ecosphere, 3, 1–12.

[ece370309-bib-0045] Pinheiro, J. , Bates, D. , & R Core Team . (2022). Nlme: Linear and nonlinear mixed effects models . R Package Version 3.1‐157, https://CRAN.R‐project.org/package=nlme

[ece370309-bib-0046] Poorter, H. , & Nagel, O. (2000). The role of biomass allocation in the growth response of plants to different levels of light, CO_2_, nutrients and water: A quantitative review. Functional Plant Biology, 27, 595–607.

[ece370309-bib-0047] Poorter, H. , Niklas, K. J. , Reich, P. B. , Oleksyn, J. , Poot, P. , & Mommer, L. (2012). Biomass allocation to leaves, stems and roots: Meta‐analyses of interspecific variation and environmental control. New Phytologist, 193, 30–50. 22085245 10.1111/j.1469-8137.2011.03952.x

[ece370309-bib-0048] R Core Team . (2022). R: A language and environment for statistical computing. R Foundation for Statistical Computing. https://www.R‐project.org/

[ece370309-bib-0049] Reynolds, H. L. , & Pacala, S. W. (1993). An analytical treatment of root‐to‐shoot ratio and plant competition for soil nutrient and light. American Naturalist, 141, 51–70. 10.1086/285460 19426022

[ece370309-bib-0050] Ross, C. W. (1974). Plant physiology laboratory manual. Wadsworth Publishing Company.

[ece370309-bib-0051] Schmidt, C. B. , Funk, J. L. , Wolf, A. A. , Akana, P. R. , Palmer, M. I. , & Menge, D. N. L. (2023). Nitrogen fixation responds to soil nitrogen availability at low but not high light in two understory species. Journal of Ecology, 111, 916–926.

[ece370309-bib-0052] Sellstedt, A. (1986). Nitrogen and carbon utilization in *Alnus incana* fixing N_2_ or supplied with NO_3_ ^−^ at the same rate. Journal of Experimental Botany, 37, 786–797.

[ece370309-bib-0053] Sellstedt, A. , & Huss‐Danell, K. (1986). Biomass production and nitrogen utilization by *Alnus incana* when grown on N_2_ or NH_4_ ^+^ made available at the same rate. Planta, 167, 387–394. 24240309 10.1007/BF00391344

[ece370309-bib-0054] Shearer, G. , & Kohl, D. H. (1986). N_2_‐fixation in field settings: Estimations based on natural ^15^N abundance. Functional Plant Biology, 13, 699–756.

[ece370309-bib-0055] Shirley, H. L. (1929). The influence of light intensity and light quality upon the growth of plants. American Journal of Botany, 16, 354–390.

[ece370309-bib-0056] Soper, F. M. , Taylor, B. N. , Winbourne, J. B. , Wong, M. Y. , Dynarski, K. A. , Reis, C. R. G. , Peoples, M. B. , Cleveland, C. C. , Reed, S. C. , Menge, D. N. L. , & Perakis, S. S. (2021). A roadmap for sampling and scaling biological nitrogen fixation in terrestrial ecosystems. Methods in Ecology and Evolution, 12, 1122–1137.

[ece370309-bib-0057] Sprent, J. I. (2009). Legume nodulation: A global perspective. Wiley Blackwell.

[ece370309-bib-0058] Taylor, B. N. , & Menge, D. N. L. (2018). Light regulates tropical symbiotic nitrogen fixation more strongly than soil nitrogen. Nature Plants, 4, 655–661. 30127409 10.1038/s41477-018-0231-9

[ece370309-bib-0059] Taylor, B. N. , & Menge, D. N. L. (2021). Light, nitrogen supply, and neighboring plants dictate costs and benefits of nitrogen fixation for seedlings of a tropical nitrogen‐fixing tree. New Phytologist, 231, 1758–1769. 34028829 10.1111/nph.17508

[ece370309-bib-0060] Thornley, J. H. (1972). A balanced quantitative model for root: Shoot ratios in vegetative plants. Annals of Botany, 36, 431–441.

[ece370309-bib-0061] Tjepkema, J. D. , & Winship, L. J. (1980). Energy requirement for nitrogen fixation in actinorhizal and legume root nodules. Science, 209, 279–281. 7384801 10.1126/science.7384801

[ece370309-bib-0062] Uni, D. , Klein, T. , Masci, T. , Winters, G. , & Sheffer, E. (2024). Strong regulation of nitrogen supply and demand in a key desert legume tree. Environmental and Experimental Botany, 224, 105823. 10.1016/j.envexpbot.2024.105823

[ece370309-bib-0063] Werner, G. D. , Cornwell, W. K. , Sprent, J. I. , Kattge, J. , & Kiers, E. T. (2014). A single evolutionary innovation drives the deep evolution of symbiotic N_2_‐fixation in angiosperms. Nature Communications, 5, 4087. 10.1038/ncomms5087 PMC4059933 24912610

[ece370309-bib-0064] Wilson, J. B. (1988). A review of evidence on the control of shoot: Root ratio, in relation to models. Annals of Botany, 61, 433–449.

[ece370309-bib-0065] Wolf, A. A. , Funk, J. L. , & Menge, D. N. L. (2017). The symbionts made me do it: Legumes are not hardwired for high nitrogen concentrations but incorporate more nitrogen when inoculated. New Phytologist, 213, 690–699. 27859292 10.1111/nph.14303

[ece370309-bib-0066] Wurzburger, N. , & Hedin, L. O. (2016). Taxonomic identity determines N_2_ fixation by canopy trees across lowland tropical forests. Ecology Letters, 19, 62–70. 26584690 10.1111/ele.12543

[ece370309-bib-0067] Zheng, M. , Zhou, Z. , Luo, Y. , Zhao, P. , & Mo, J. (2019). Global pattern and controls of biological nitrogen fixation under nutrient enrichment: A meta‐analysis. Global Change Biology, 25, 3018–3030. 31120621 10.1111/gcb.14705

