# Supplementary material for: Nitrogen fixation and fertilization have similar effects on biomass allocation in nitrogen‐fixing plants
Source: Ecol Evol. 2024 Sep 17;14(9):e70309. doi: 10.1002/ece3.70309 (PMC11407827; doi:10.1002/ece3.70309)
Supplement: Supplementary file 1 — Data S1. [file ECE3-14-e70309-s001.docx]

**Supporting Information**

**Nitrogen fixation and fertilization have similar effects on biomass allocation in nitrogen-fixing plants**

**Note S1**

Here we show results from the alternate analyses where we used the rate of N fixation (N fixed g C^–1^ y^–1^) rather than the percent of N derived from N fixation (%N_dfa_) as a driver variable. Most effects are qualitatively the same as and quantitatively similar to the analogous analyses shown in the main text. As such, we will focus on comparisons to the analyses in the main text rather than a full text description of the results. The *P* values for both analyses are in Tables 1, 2, S3, and S4.

*Biomass*

For the plants grown at Barnard, the fixed effects from the mixed model were

$$Barnard rhizobial tree total biomass \left( mg \right)=1239+\boldsymbol{29.3*N}-170*I+\boldsymbol{17.2*t}\boldsymbol{N}_{\boldsymbol{dfa}}+\boldsymbol{208*P}+4.04*N*I+0.113*N*tN_{dfa}+82.1*P*I-0.795*P*tN_{dfa}$$

For the plants grown at UC Davis, the fixed effects from the mixed model were

$$UC Davis rhizobial tree seedling total biomass \left( mg \right)=305+\boldsymbol{220*N}+2198*I+\boldsymbol{22.8*t}\boldsymbol{N}_{\boldsymbol{dfa}}-60.9*P+\boldsymbol{130*N*I}+0.702*N*tN_{dfa}-181*P*I+3.39*P*tN_{dfa}$$

$$UC Davis actinorhizal tree seedling total biomass \left( mg \right)=-983+\boldsymbol{569*N}+1340*I+\boldsymbol{193*t}\boldsymbol{N}_{\boldsymbol{dfa}}+184*P-37*N*I+\boldsymbol{5.55*N*t}\boldsymbol{N}_{\boldsymbol{dfa}}+294*P*I-15.8*P*tN_{dfa}$$

$$UC Davis soybean total biomass \left( mg \right)=703+\boldsymbol{21.1*N}+175*I+\boldsymbol{1.65*t}\boldsymbol{N}_{\boldsymbol{dfa}}+4.43*P+2.58*N*I+0.0393*N*tN_{dfa}+11.9*P*I-0.743*P*tN_{dfa}$$

where all variables are as in the main text except for *tN_dfa_*, which is the plant’s N fixation rate in g N fixed g C^–1^ y^–1^. These results are qualitatively similar to the model in the main text, with the following two exceptions. For rhizobial trees at UC Davis, the biomass model with N fixed g C^–1^ y^–1^ had a significant synergistic interaction between N fertilization and inoculation, whereas in the biomass model with %N_dfa_, there was a significant synergistic interaction between N fertilization and %N_dfa_. For actinorhizal trees at UC Davis, the inoculation effect was not significant in the biomass model with N fixed g C^–1^ y^–1^, whereas it was for the biomass model with %N_dfa_.

*Belowground vs. aboveground allocation*

The fixed effects from the mixed models were

$$Barnard rhizobial tree belowground biomass \left( \% of total biomass \right)=45.4-\boldsymbol{0.154}*N-1.48*I-\boldsymbol{0.0986}*tN_{dfa}-\boldsymbol{0.337}*P+0.174*\ln\left( Biomass \right)+0.0677*N*I+0.00053*N*tN_{dfa}-0.230*P*I+0.00422*P*tN_{dfa}$$

$$UC Davis plant belowground biomass \left( \% of total biomass \right)=52.3-\boldsymbol{0.127*N}-\boldsymbol{9.68*I}-\boldsymbol{0.0251*t}\boldsymbol{N}_{\boldsymbol{dfa}}+0.165*P-\boldsymbol{0.806*}\ln\left( \boldsymbol{Biomass} \right)-15.3*S-2.32*A+\boldsymbol{0.154*N*I}-0.00028*N*tN_{dfa}-0.0810*N*S+0.0222*N*A-0.0630*P*I-0.00229*P*tN_{dfa}-0.0652*P*S+0.0513*P*A$$

As we had hypothesized (H1a), plants in both locations allocated relatively less belowground when they had more N, either from fertilization or from N fixation. At Barnard, the average uninoculated rhizobial tree seedling allocated 46% (the same % as in the analysis with %N_dfa_) belowground at our lowest N fertilization level, compared to 35% (same) at the highest N level. The effect of N fixation was similar to the effect of N fertilization. The average inoculated rhizobial tree seedling at low N allocated 45% (same) belowground if it was not fixing, compared to 30% belowground at the maximum N fixation rate (compared to 35% at 100% N_dfa_ in the analysis with %N_dfa_, though note that the maximum %N_dfa_ at Barnard was 92%, not 100%, so comparing the maximum N fixation rate to 100% N_dfa_ is not apples to apples). The two effects did not interact for the Barnard plants, so at high N, the average inoculated rhizobial tree seedling allocated 39% (same) belowground if it was not fixing, compared to 30% belowground at the maximum N fixation rate (compared to 29% at 100% N_dfa_).

Just like the N effects on biomass, the N effects on aboveground vs. belowground biomass allocation were similar in direction but more drastic in magnitude at UC Davis compared to Barnard. At UC Davis, the average uninoculated rhizobial tree seedling allocated 46% (44% for actinorhizals, compared to 49% and 46% in the analysis with %N_dfa_) belowground at the lowest N level and 39% (37% for actinorhizals, compared to 32% and 31% in the analysis with %N_dfa_) at the highest N level. Fixation had at least as large an effect as N fertilization. The average inoculated rhizobial tree seedling at low N allocated 36% (33% for actinorhizals, compared to 49% and 46%) belowground if it was not fixing, compared to 29% if it was fixing at its maximal rate (27% for actinorhizals; 28% and 26% at 100% N_dfa_). At the highest N level, the average inoculated plant allocated 37% (36% for actinorhizals, compared to 39% and 38% in the analysis with %N_dfa_) belowground if it was not fixing, whereas it allocated 27% (26% for actinorhizals, compared to 23% and 21%) belowground when it was fixing at its maximum rate. Furthermore, N fixation levels were higher at UC Davis: an average of 100 mg N fixed g C^–1^ y^–1^ (69 %N_dfa_) for rhizobial tree seedlings at UC Davis compared to 27 mg N fixed g C^–1^ y^–1^ (24% N_dfa_) at Barnard. For actinorhizal tree seedlings, average N fixation rates at were 37 mg N fixed g C^–1^ y^–1^ (54 %N_dfa_). Therefore, the large effects at UC Davis were even stronger than they appear in the coefficients: the effects on belowground allocation were at least as large per unit N fixed, but their realized effects were even larger because the plants were fixing more N.

*Leaves vs. stem allocation*

The fixed effects from the mixed models were

$$Barnard rhizobial tree biomass \left( \% of aboveground biomass \right)=72.3+0.0379*N-1.98*I+\boldsymbol{0.109*t}\boldsymbol{N}_{\boldsymbol{dfa}}+0.102*P-\boldsymbol{2.29*}\ln\left( \boldsymbol{Biomass} \right)+0.0181*N*I+0.00015*N*tN_{dfa}-0.124*P*I+0.00151*P*N_{dfa}$$

$$UC Davis plant foliar biomass \left( \% of aboveground biomass \right)=62.3-0.0350*N+\boldsymbol{3.02*I}+\boldsymbol{0.0828*t}\boldsymbol{N}_{\boldsymbol{dfa}}-\boldsymbol{0.465*P}-\boldsymbol{1.29*}\ln\left( \boldsymbol{Biomass} \right)-15.7*S+13.0*A-\boldsymbol{0.179*N*I}-0.000089*N*tN_{dfa}+\boldsymbol{0.687*N*S}+0.0424*N*A+0.404*P*I-0.00002*P*tN_{dfa}+0.0259*P*S+0.111*P*A$$

These results were qualitatively the same as in the analysis with %N_dfa_ except that the inoculation effect was significant in Barnard in the main text, the inoculation effect was not significant in the main text in UC Davis, and the N fertilization by N fixation interaction was significant in the main text.

Table S1. Symbiotic nitrogen fixing species grown in this experiment

| Species^1^ | Habitat | Barnard | UC Davis | Seed source | Inoculum source | Inoculum type at UC Davis^2^ |
| --- | --- | --- | --- | --- | --- | --- |
| *Robinia pseudoacacia*^R^ | Temp | 2016,7 | 2018 | Sheffields | New York, USA | Culture from crushed nodules |
| *Alnus rubra*^A^ | Temp |  | 2018 | Sheffields | Oregon, USA | Both culture and slurry |
| *Gliricidia sepium*^R^ | Trop | 2017 | 2018 | Sheffields | Florida, USA | Culture from crushed nodules |
| *Casuarina equisetifolia*^A^ | Trop |  | 2018 | Hawaii, USA | Hawaii, USA | Crushed nodule slurry |
| *Acacia koa*^R^ | Trop | 2016,7 | 2018 | Ebay | Hawaii, USA | Both culture and slurry |
| *Morella faya*^A^ | Trop |  | 2018 | Hawaii, USA | Hawaii, USA | Crushed nodule slurry |
| *Acacia farnesiana*^R^ | Temp | 2016 | 2018 | Sheffields | Florida, USA | Culture from crushed nodules |
| *Elaeagnus angustifolia*^A^ | Temp |  | 2018 | Sheffields | New York, USA | Crushed nodule slurry |
| *Enterolobium cyclocarpum*^R^ | Trop | 2016 | 2018 | Banana Tree | Mexico | Culture from crushed nodules |
| *Leucaena leucocephala*^R^ | Trop | 2016 |  | Sheffields | Florida, USA | Not grown at UC Davis |
| *Albizia julibrissin*^R^ | Temp | 2016 |  | Banana Tree | Florida, USA | Not grown at UC Davis |
| *Sophora chrysophylla*^R^ | Trop | 2016 |  | Hawaii, USA | Hawaii, USA | Not grown at UC Davis |
| *Alnus acuminata*^A^ | Trop |  | 2018 | Sheffields | Mexico | Both culture and slurry |
| *Morella cerifera*^A^ | Trop |  | 2018 | Hobby Seeds | Florida, USA | Crushed nodule slurry |
| *Glycine max* (soybean)^R^ | Ag |  | 2018 | Johnny’s Seeds | Commercial verdesian GUARD-N | Commercial inoculant |

^1^R indicates rhizobial (legume plant, *Rhizobia*-type bacteria), A indicates actinorhizal (non-legume plant, *Frankia*-type bacteria)

^2^All plants grown at Barnard used crushed nodule slurries.

Table S2. Growing conditions for each symbiotic nitrogen fixing species

| Species^1^ | Days | N levels | Low N^2^ | High N^2^ | Low P^2^ | High P^2^ | Treatment Start | Harvest Date | n_uninoc_^3^ | n_inoc_^3^ |
| --- | --- | --- | --- | --- | --- | --- | --- | --- | --- | --- |
| *Robinia pseudoacacia*^R^ 2016 | 237 | 9 | 0.3 | 30 | 0.34 | 15 | 10/21/2015 | 6/14/2016 | 26 | 29 |
| *Robinia pseudoacacia*^R^ 2017 | 148-162 | 9 | 0.3 | 75 | 0.17 | 15 | 10/18/2016 | 3/15-3/29/2017 | 5 | 30 |
| *Robinia pseudoacacia*^R^ 2018 | 125-147 | 9 | 0.3 | 60 | 0.17 | 15 | 11/22/2017 | 3/26-4/18/2018 | 28 | 30 |
| *Alnus rubra*^A^ 2018 | 278-300 | 9 | 0.3 | 60 | 0.17 | 15 | 12/21/2017 | 9/25-10/16/2018 | 15 | 27 |
| *Gliricidia sepium*^R^ 2017 | 106-114 | 9 | 0.3 | 75 | 0.17 | 15 | 4/12/2017 | 7/27-8/4/2017 | 30 | 30 |
| *Gliricidia sepium*^R^ 2018 | 119-126 | 9 | 0.3 | 60 | 0.17 | 15 | 1/18/2018 | 5/17-5/24/2017 | 28 | 30 |
| *Casuarina equisetifolia*^A^ 2018 | 304-338 | 9 | 0.3 | 60 | 0.17 | 15 | 1/19/2018 | 11/19-12/12/2018 | 29 | 30 |
| *Acacia koa*^R^ 2016 | 182 | 9 | 0.3 | 30 | 0.34 | 15 | 9/1/2015 | 3/1/2016 | 24 | 30 |
| *Acacia koa*^R^ 2017 | 93-99 | 9 | 0.3 | 75 | 0.17 | 15 | 4/18/2017 | 7/20-7/26/2017 | 30 | 30 |
| *Acacia koa*^R^ 2018 | 308-322 | 9 | 0.3 | 60 | 0.17 | 15 | 12/21/2017 | 10/25-11/8/2018 | 16 | 27 |
| *Morella faya*^A^ 2018 | 169-177 | 6 | 3.3 | 60 | 0.17 | 15 | 7/5/2018 | 12/21-12/29/2018 | 4 | 14 |
| *Acacia farnesiana*^R^ 2016 | 190 | 9 | 0.3 | 30 | 0.34 | 15 | 11/24/2015 | 6/1/2016 | 28 | 27 |
| *Acacia farnesiana*^R^ 2018 | 179-186 | 9 | 0.3 | 60 | 0.17 | 15 | 1/18/2018 | 7/16-7/29/2018 | 28 | 30 |
| *Elaeagnus angustifolia*^A^ 2018 | 175-190 | 9 | 0.3 | 60 | 0.17 | 15 | 1/2/2018 | 6/26-7/11/2018 | 13 | 29 |
| *Enterolobium cyclocarpum*^R^ 2016 | 145 | 9 | 0.3 | 30 | 0.34 | 15 | 11/4/2015 | 3/28/2016 | 26 | 30 |
| *Enterolobium cyclocarpum*^R^ 2018 | 271-277 | 9 | 0.3 | 60 | 0.17 | 15 | 3/16/2018 | 12/12-12/18/2018 | 23 | 28 |
| *Leucaena leucocephala*^R^ 2016 | 121-129 | 9 | 0.3 | 75 | 0.17 | 15 | 7/12/2016 | 11/10-11/18/2016 | 30 | 30 |
| *Albizia julibrissin*^R^ 2016 | 99-106 | 9 | 0.3 | 75 | 0.17 | 15 | 8/16/2016 | 11/23-30/2016 | 30 | 30 |
| *Sophora chrysophylla*^R^ 2016 | 149-155 | 9 | 0.3 | 75 | 0.17 | 15 | 7/12/2016 | 12/7-14/2016 | 28 | 30 |
| *Alnus acuminata*^A^ 2018 | 288-291 | 9 | 0.3 | 60 | 0.17 | 15 | 3/16/2018 | 12/29/2018-1/1/2019 | 17 | 19 |
| *Morella cerifera*^A^ 2018 | 166-169 | 9 | 0.3 | 60 | 0.17 | 15 | 7/5/2018 | 12/18-12/21/2018 | 21 | 29 |
| *Glycine max* (soybean)^R^ 2018 | 42-43 | 9 | 0.3 | 60 | 0.17 | 15 | 9/27/2018 | 11/8-11/9/2018 | 30 | 30 |

^1^R indicates rhizobial (legume plant, *Rhizobia*-type bacteria), A indicates actinorhizal (non-legume plant, *Frankia*-type bacteria)

^2^Fertilization flux in g N or P m^−2^ pot y^−1^

^3^n_uninoc_ and n_inoc_ are the numbers of plants used in the analysis for inoculated and uninoculated individuals, respectively.

Table S3. Significance of fixed effect coefficients for statistical models that use the N fixation rate (g N g C^–1^ y^–1^; written here as *tN_dfa_*) instead of %N_dfa_ as a driver

|  | $\beta_{N}$* | $\beta_{I}$ | $\beta_{tN_{dfa}}$ | $\beta_{P}$ | $\beta_{A}$ | $\beta_{S}$ | $\beta_{\ln\left( B \right)}$ | $\beta_{N\times I}$ | $\beta_{N\times tNdfa}$ | $\beta_{N\times A}$ | $\beta_{N\times S}$ | $\beta_{P\times I}$ | $\beta_{P\times tNdfa}$ | $\beta_{P\times A}$ | $\beta_{P\times S}$ |
| --- | --- | --- | --- | --- | --- | --- | --- | --- | --- | --- | --- | --- | --- | --- | --- |
| Barnard rhizobial tree total biomass (mg) | **<.0001** | –.5838 | **.0006** | **<.0001** |  |  |  | .6776 | .7570 |  |  | .1088 | –.6546 |  |  |
| UC Davis rhizobial tree total biomass (mg) | **<.0001** | .*0901* | **.0074** | –.6350 |  |  |  | **.0070** | *.0587* |  |  | –.4306 | .*0688* |  |  |
| UC Davis actinorhizal tree total biomas (mg) | **<.0001** | .6504 | **<.0001** | .5689 |  |  |  | –.7408 | **.0264** |  |  | ,6476 | –.3211 |  |  |
| UC Davis soybean total biomass (mg) | **<.0001** | .1318 | **.0005** | .7299 |  |  |  | .5300 | .3362 |  |  | ,5772 | –*.0899* |  |  |
| Barnard belowground biomass (% of total) | –**<.0001** | –.3173 | –**<.0001** | –**.0356** |  |  | .7421 | .1432 | .7644 |  |  | –.3457 | .6224 |  |  |
| UC Davis belowground biomass (% of total) | –**.0032** | –**<.0001** | –**.0038** | .3398 | –.6843 | –.1574 | –.**0291** | **.0009** | –.5606 | .6110 | –.2186 | –.7850 | –.3662 | .8058 | –.8431 |
| Barnard foliar biomass (% of aboveground) | .2621 | –.1968 | **<.0001** | .5384 |  |  | –**<.0001** | .7056 | .9344 |  |  | –.6245 | .8649 |  |  |
| UC Davis foliar biomass (% aboveground) | –.4794 | **.0370** | **<.0001** | –**.0198** | .0595 | –.1819 | –**.0024** | **–.0009** | –.8809 | .3987 | **<.0001** | *.*1297 | –.9953 | .6444 | .9457 |

**P* values are shown as negative when the effect is negative. *P* values are shown in bold when they are < 0.05 and in italics when they are between 0.05 and 0.1. The coefficient names correspond to driver variables *N* (N supplied as fertilizer), *I* (inoculation), *tN_dfa_* (the rate of the plant’s N fixation), *P* (P supplied as fertilizer), *A* (actinorhizal tree as opposed to rhizobial tree), *S* (soybean as opposed to rhizobial tree), ln(*B*) (the natural logarithm of biomass), and interactions between some pairs of these variables.

Table S4. Random intercepts for statistical models that use the N fixation rate (g N g C^–1^ y^–1^) instead of %N_dfa_ as a driver. Some species were grown in both greenhouses, whereas others were only grown in one (Barnard or UC Davis).

|  | Barnard | | | | UC Davis | | | |
| --- | --- | --- | --- | --- | --- | --- | --- | --- |
| Species | Biomass (mg) | Belowground (% of total) | Foliar (% of aboveground) |  | Biomass (mg) | Belowground (% of total) | Foliar (% of aboveground) |  |
| *Acacia farnesiana* | 1067 | 44.4 | 61.5 |  | 1456 | 51.0 | 52.9 |  |
| *Acacia koa* | 976 | 29.9 | 81.2 |  | 869 | 46.1 | 66.8 |  |
| *Alnus acuminata* |  |  |  |  | 4450 | 60.3 | 64.4 |  |
| *Albizia julibrissin* | –480 | 61.0 | 81.4 |  |  |  |  |  |
| *Alnus rubra* |  |  |  |  | –1006 | 69.1 | 53.6 |  |
| *Casuarina equisetifolia* |  |  |  |  | 4694 | 56.0 | 70.2 |  |
| *Elaeagnus angustifolia* |  |  |  |  | –1361 | 44.3 | 42.3 |  |
| *Enterolobium cyclocarpum* | 4119 | 39.6 | 55.6 |  | –629 | 52.7 | 64.8 |  |
| *Glycine max* (soybean) |  |  |  |  | N/A | 52.3 | 62.3 |  |
| *Gliricidia sepium* | 952 | 40.5 | 64.4 |  | 215 | 56.7 | 61.3 |  |
| *Leucaena leucocephala* | 2272 | 66.6 | 73.4 |  |  |  |  |  |
| *Morella cerifera* |  |  |  |  | –6520 | 42.9 | 70.6 |  |
| *Morella faya* |  |  |  |  | –6158 | 41.0 | 72.3 |  |
| *Robinia pseudoacacia* | 1351 | 40.4 | 78.1 |  | –396 | 54.9 | 65.4 |  |
| *Sophora chrysophylla* | –348 | 40.5 | 82.3 |  |  |  |  |  |

Supplementary Figures

Figure S1. Biomass and biomass allocation for seedlings of *Acacia farnesiana* across an N fertilization gradient for Barnard species. Details as in Fig. 1 except that only one species is shown.

Figure S2. Biomass and biomass allocation for seedlings of *Acacia koa* across an N fertilization gradient for Barnard species. Details as in Fig. 1 except that only one species is shown.

Figure S3. Biomass and biomass allocation for seedlings of *Albizia julibrissin* across an N fertilization gradient for Barnard species. Details as in Fig. 1 except that only one species is shown.

Figure S4. Biomass and biomass allocation for seedlings of *Enterolobium cyclocarpum* across an N fertilization gradient for Barnard species. Details as in Fig. 1 except that only one species is shown.

Figure S5. Biomass and biomass allocation for seedlings of *Gliricidia sepium* across an N fertilization gradient for Barnard species. Details as in Fig. 1 except that only one species is shown.

Figure S6. Biomass and biomass allocation for seedlings of *Leucaena leucocephala* across an N fertilization gradient for Barnard species. Details as in Fig. 1 except that only one species is shown.

Figure S7. Biomass and biomass allocation for seedlings of *Robinia pseudoacacia* across an N fertilization gradient for Barnard species. Details as in Fig. 1 except that only one species is shown.

Figure S8. Biomass and biomass allocation for seedlings of *Sophora chrysophylla* across an N fertilization gradient for Barnard species. Details as in Fig. 1 except that only one species is shown.
